# ReactionDataExtractor 2.0: A Deep Learning Approach for Data Extraction from Chemical Reaction Schemes

**DOI:** 10.1021/acs.jcim.3c00422

**Published:** 2023-09-20

**Authors:** Damian M. Wilary, Jacqueline M. Cole

**Affiliations:** † Cavendish Laboratory, Department of Physics, University of Cambridge, J. J. Thomson Avenue, Cambridge, CB3 0HE, U.K.; ‡ ISIS Neutron and Muon Source, STFC Rutherford Appleton Laboratory, Harwell Science and Innovation Campus, Didcot, Oxfordshire OX11 0QX, U.K.

## Abstract

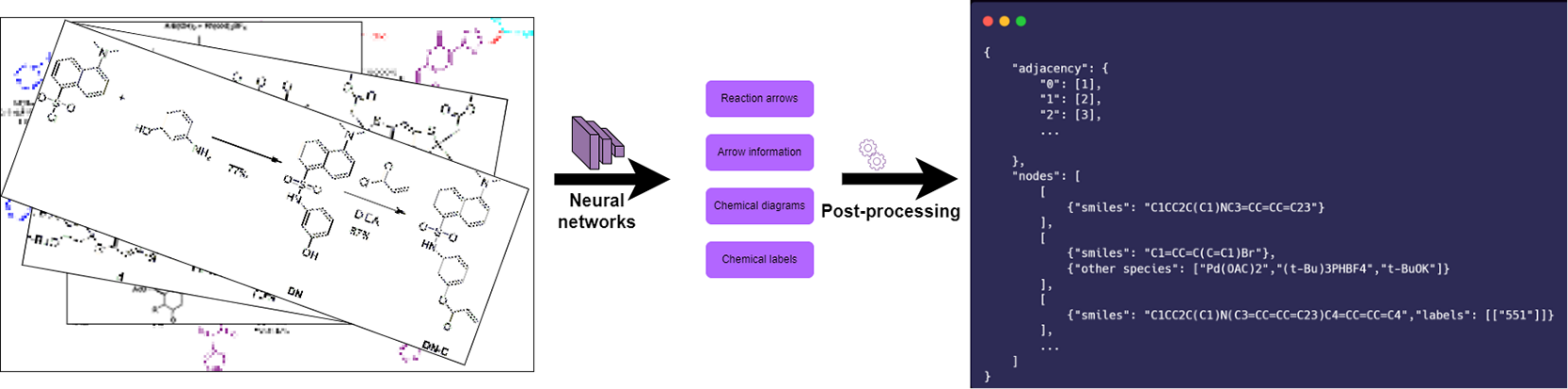

Knowledge in the chemical domain is often disseminated graphically via chemical reaction schemes. The task of describing chemical transformations is greatly simplified by introducing reaction schemes that are composed of chemical diagrams and symbols. While intuitively understood by any chemist, like most graphical representations, such drawings are not easily understood by machines; this poses a challenge in the context of data extraction. Currently available tools are limited in their scope of extraction and require manual preprocessing, thus slowing down the speed of data extraction. We present a new tool, ReactionDataExtractor v2.0, which uses a combination of neural networks and symbolic artificial intelligence to effectively remove this barrier. We have evaluated our tool on a test set composed of reaction schemes that were taken from open-source journal articles and realized F1 score metrics between 75 and 96%. These evaluation metrics can be further improved by tuning our object-detection models to a specific chemical subdomain thanks to a data-driven approach that we have adopted with synthetically generated data. The system architecture of our tool is modular, which allows it to balance speed and accuracy to afford an autonomous, high-throughput solution for image-based chemical data extraction.

## Introduction

Research in the materials-science community is becoming more data driven than ever. Thanks to initiatives such as the Harvard Clean Energy Project^1^ and Novel Materials Discovery (NOMAD),^2^ computational data are accessible for researchers around the world to drive the development of new materials. The availability of millions of data concerning the structures of materials and their cognate properties enables so-called big-data approaches in science. Contemporary approaches for data mining often use natural language processing (NLP) to extract data from text sources.^3−12^

Computer vision, another important branch of AI, also has great potential in the area of mining image-based chemical information from scientific documents. In the field of synthetic chemistry, a myriad of chemical reaction schemes are displayed in the literature. Extraction of data from these sources is the focus of the presented work. When data extraction from a chemical reaction scheme is considered, the main goal is to accurately find and classify its individual elements. In the computer-vision domain, this is known as object detection, and it is a well-researched task. Thereby, a wide range of detection networks exists, depending on usage domain, and required inference speed and accuracy.^13−25^

Computer vision for optical chemical structure recognition (OCSR) is a core challenge that one needs to resolve if one is to automate the interpretation of image-based chemical reaction schemes; chemical diagrams are ubiquitous in such schemes. Computer vision has been applied to interpret chemical diagrams in images for around 30 years^26^ and the problem of converting raster images of chemical diagrams into digital formats, e.g., machine-readable format such as a simplified molecular-input line-entry system (SMILES),^27^ has been well studied. Initial attempts^26,28−31^ at tackling this problem included rule-based algorithms which involve defining primitives that form carbon skeletons, bonds, and superatoms. However, such tools offer a limited scope for improving their assessment metrics, owing to their complex and rigid nature. More modern solutions^32−35^ are driven by data, and use neural networks to approximate highly nonlinear mappings from the signal domain to output digitized representations of chemical information. A recently published paper on the DECIMER^34^ software, which employs a transformer-based architecture, affords an accuracy of 90% (measured from the average Tanimoto similarity). An OCSR-based tool, ChemSchematicResolver, developed by Beard and Cole^36^ is also capable of separating chemical diagrams and their labels into two classes where they appear together in chemical schematics; however, its usage is limited to images that contain only these two types of objects.

Few solutions currently exist to extract data from full chemical reaction schemes, which represents a substantial extension to the OCSR problem. In addition to the need to recognize chemical diagrams from images, chemical reaction schemes involve object-detection problems in resolving chemical structures and other reaction descriptors, as well as making connections between different parts of the reaction scheme (e.g., diagrams and their labels) and establishing context for the chemical transformations by deciphering the role of each chemical displayed in different steps of a chemical reaction (e.g., reactant, intermediate, product) and making logical connections between them. Qian et al.^38^ have recently formulated a method of data extraction from reaction schemes via an image-to-sequence translation task. Their model is a single encoder-decoder architecture closely following Pix2Seq,^39^ which has been developed as a generic object detection model, whereby the task is expressed in the language modeling domain. This formulation allows extension of object detection into an ordered sequence detection that is suitable for reaction scheme parsing. Qian et al.^38^ mentioned further possible improvements of their methodology via use of more annotated data.

We present a tool, ReactionDataExtractor v2.0, that is capable of capturing additional data such as the reaction arrows, arrow annotation information (environmental conditions of reactions) placed below and above reaction arrows, and chemical labels that may form an important context surrounding the chemical diagrams. Our tool can be used on reaction schemes from a variety of sources, e.g., journal articles. Extracting chemical labels and linking them to their parent chemical diagrams using our tool allow the identification of chemical species which are often mentioned in the main text using merely their labels. Furthermore, our work is also complementary to the OCSR approaches which analyze individual chemical diagrams, whereas our tool infers relationships between the different chemical diagrams based on spatial information, as well as other visual information (e.g., reaction arrows), thus leading to the reconstruction of full reaction schemes in a machine-readable format. Previous attempts, such as ReactionDataExtractor v1.0^37^ are more limited in scope owing to the assumptions that the tool makes about elements of the reaction schemes. For example, its use of the Hough transform for arrow detection limits the scope of resolving simple reaction schemes to those with solid arrows only, while its diagram-extraction model makes implicit assumptions about the presence and length of a carbon skeletal backbone in chemical diagrams. Therefore, prior to data extraction, a manual filtering step is required in ReactionDataExtractor version 1.0 to align the data distribution to its defined scope, thus limiting its practical use for high-throughput data extraction. This paper presents v2.0, which has been designed to overcome these two limitations. Thanks to the use of deep-learning approaches for object detection, neural-network-based models have become more flexible in accommodating a larger variety of input data. The system architecture of ReactionDataExtractor version 2.0 combines neural-network models with new symbolic algorithms that aim to inject expert knowledge into the pipeline. This new foundational architecture of ReactionDataExtractor effectively lifts the barriers to extraction that v1.0 encountered. Furthermore, the data required to train the neural network are generated synthetically (artificially); thus, its data-extraction process can be fine-tuned to a specific chemical subdomain by training neural-network models on data that were synthetically generated according to the user-specified schema. This makes the tool more suited for the automatic generation of databases of chemical reactions.

## System Overview

**Figure 1:**
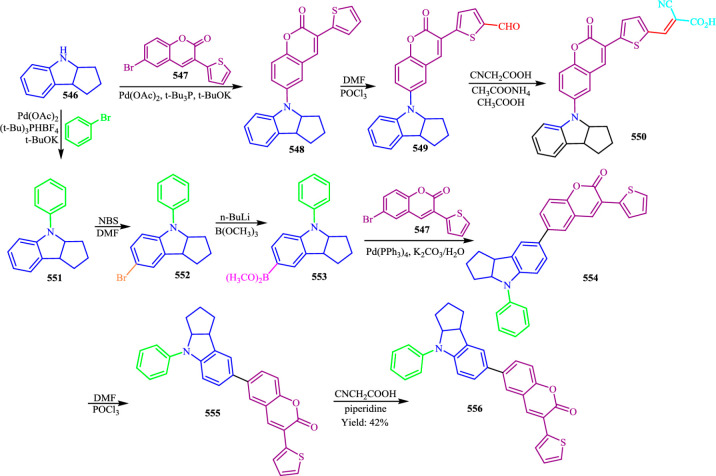
Example reaction scheme^40^ that will serve as the working example in this paper to illustrate the key steps of the operational pipeline in ReactionDataExtractor v2.0. No changes were made by the authors to this originally published reaction scheme.

ReactionDataExtractor is used for automatic data extraction from images of chemical reaction schemes. An example of a reaction scheme is shown in Figure 1, which we shall use as the working example throughout this paper to illustrate certain distinct features of our tool.

**Figure 2:**
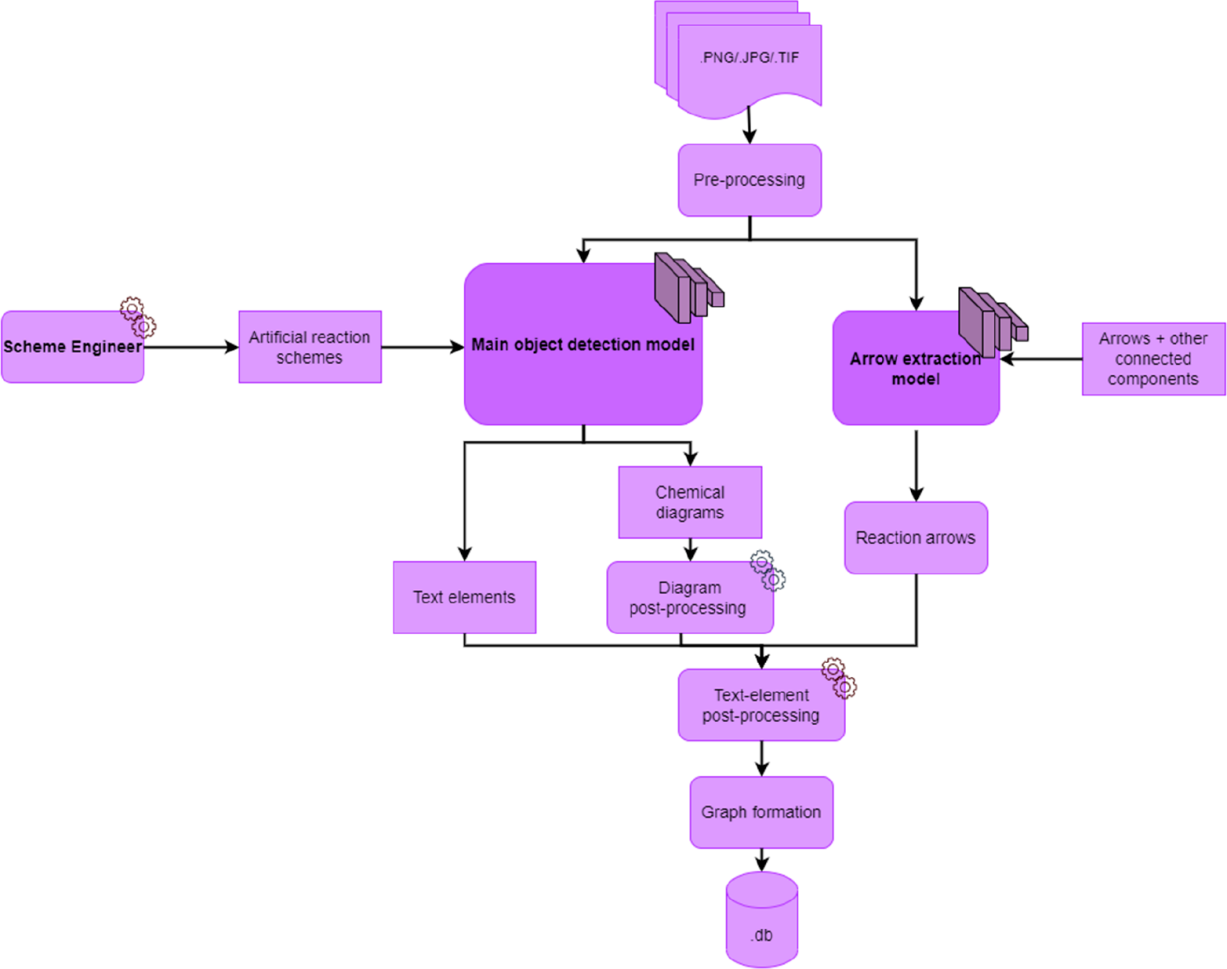
System architecture of ReactionDataExtractor v2.0 with its operational pipeline being shown as propagating from top to bottom and its synthetic data-generation pipeline being shown across a horizontal traverse. The block-set symbols denote processes that use neural networks, and the cog symbols highlight symbolic postprocessing and data-generation algorithms.

ReactionDataExtractor v2.0 comprises two parts: its synthetic data-generation pipeline (Scheme Engineer) and its main operational pipeline. The relationship between the two is shown in Figure 2.

## Synthetic Data-Generation Pipeline

### A “Scheme Engineer” Workflow: Conceptual Idea and Rationale for Development

**Figure 3:**
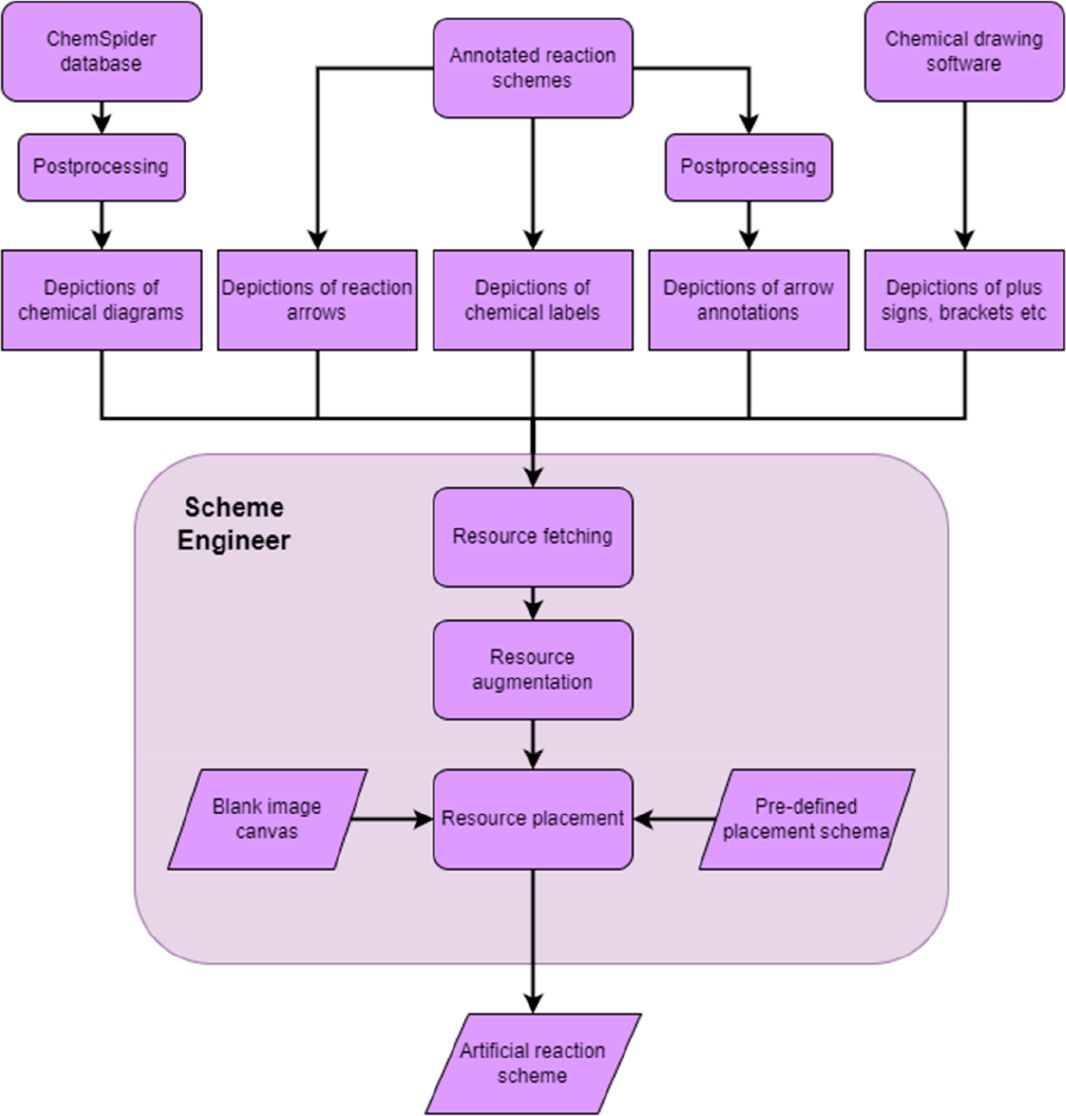
Synthetic data generation process. In order to train our main detection model, we created artificial reaction schemes. This is done by first obtaining the visual data components of a reaction scheme from various sources depending on data type and then using these data as resources to populate an a priori blank image canvas. Placement of these resources is governed by a user-defined placement schema.

Even though chemical reaction schemes can convey unique semantic information, they generally obey common types of schema. For example, simple chemical reaction schemes tend to be drawn horizontally along a single line, with arrows defining reaction steps and separating reactants and products in each steps. A good degree of order is generally displayed in such schemes, from which chemical patterns emerge. It is therefore possible to recreate the data distribution via the means of a so-called synthetic data-generation process, whereby the data resembling real-world data are created artificially. To achieve this goal, we need to carefully define the schema and populate the schemes using elements of individual reaction schemes. To this end, we have developed a pipeline called the Scheme Engineer. The process of creating these artificial reaction scheme data is visualized in Figure 3. In the context of this generation process, we use terms “synthetic data generation” to represent the general concept of our design and ‘artificial reaction schemes’ to denote the created data. This is to provide more clarity as the word “synthetic” has a special meaning in the field of chemistry, and its improper use can lead to confusion.

In the next section, we describe in detail how the individual elements (chemical diagrams, labels, reaction arrows, and their annotations) of reaction schemes are sourced. Later, we describe how these elements are used and augmented to create new, artificial reaction schemes.

### Sourcing Data Components to Generate Artificial Reaction Schemes

In this section, we describe how the individual data components (chemical diagrams, reaction arrows and their annotations, chemical labels, etc.) are sourced and preprocessed for use in order to generate artificial reaction schemes for training the main object detection model. The initial data used to generate the individual pieces of our artificial reaction schemes were sourced from publicly available databases as well as from a small number (about 150) of annotated reaction schemes. The reaction schemes were taken from closed-access articles from scientific journals. The closed-access criterion ensured that there is no intersection between these data and our evaluation set, described later in the technical evaluation section. These annotations were used indirectly to train the main object-detection model as well as directly to train the arrow-detection model.

To obtain examples of individual chemical diagrams that form pieces of the artificial reaction schemes, we randomly chose 10,000 chemical schematics that are rendered in the ChemSpider^41^ database. A dilation with a large disk-shaped kernel was applied to each image to ensure that a single chemical species and all connected components belonging to the largest dilated region were selected to represent the chemical molecule. Examples of arrow annotations and chemical labels that form pieces of our artificial reaction schemes were taken directly from the annotated images. We extracted regions of these images that contain 418 arrow annotations and 951 chemical labels. We postprocessed the cropped arrow annotations to remove fragments of arrows. We also manually created a small number of plus signs, brackets, and similar text symbols to provide negative samples that could inform the object-detection model. It is important to note that the choice of all reaction elements from this assembled set of reaction elements, including chemical diagrams, is completely random. Therefore, the artificial reaction schemes that are afforded by this Scheme Engineer process make no chemical sense, which is a potential limitation of this method. Nevertheless, the image-based features of the individual elements and relationships between the elements appear to be sufficient for the model to learn meaningful representations, which is reflected in high evaluation metrics reported later.

### Artificial Scheme Generation

**Figure 4:**
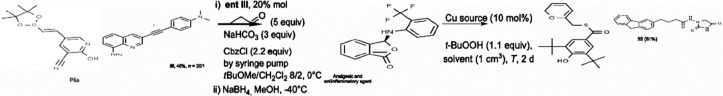
Example of an artificial reaction scheme. From a chemical point of view, this “scheme” makes no sense, but this is not the goal; rather, the imagery needs to contain important diagram and text-element features, as well as spatial relationships between them; the features are not chemically related, so an ensemble chemical interpretation is not appropriate. This scheme was generated by using the linear schema described above.

In this section, we describe how the components of reaction schemes described earlier are used to create new artificial reaction schemes. The schemes are created by placing the imagery for chemical diagrams, their reaction arrows, chemical labels, and arrow annotations on an empty image-based canvas according to a predefined schema, which directs both the absolute and relative positions of individual scheme elements. These schemas are defined by the user and specify the general layout of a reaction scheme. For example, a linear schema guides the creation of simple reaction schemes, which can be drawn along a single line, while a cyclic schema allows creation of schemes that resemble simple catalytic cycles. The schemes also define how many reaction steps are allowed, how many diagrams can be placed per step, and how many chemical diagrams and arrows should have labels and arrow annotations, respectively. Prior to the placement of the image of each reaction component onto the canvas, it undergoes an augmentation process. Thereby, each image is subjected to affine transformations (translations, scaling, rotations) and is randomly blurred to make the object-detection neural network more robust to dealing with low-resolution images since high-resolution input data are not always readily available. We also add a small random number of negative samples to assist in the object-detection model training. We used these artificially generated data to train this model. An example of an artificial reaction scheme is given in Figure 4. This method allows us to produce large amounts of data within the modeled data distribution. Furthermore, we can define more layouts using a user-defined schema. From a practical point of view, potential limitations are those associated with any synthetic data generation process and data augmentation. These tools work well when the data distribution is well-defined but scheme patterns outside of the distribution might not be easily handled. User-defined schema are one method to overcome this; nevertheless, it is challenging or even impossible to define all patterns. Furthermore, in its current form, there is still room for improvement in some areas, e.g., the relative position of the reaction arrow and its annotation, which might affect performance.

## Main Operational Pipeline

### Scheme Extraction Pipeline

The main extraction pipeline is summarized in Figure 2. After preprocessing, an input image undergoes the core extraction process. The image is fed to an object-detection model which detects three classes of chemical information: chemical diagrams, chemical labels, and arrow annotations. The fourth semantically important class, reaction arrows, is extracted in a parallel step. To achieve this, connected components (CCs) are filtered according to simple criteria to yield proposals that are then fed to an arrow-detection model. Initial experiments showed that a single object-detection model performed well for three of the classes of chemical information but afforded poor results for reaction arrows. This exception is likely due to the very small size of the arrows and the little semantic information that is conveyed by their constituent shapes, as opposed to the larger and/or more semantically complex chemical diagrams, arrow annotations, and chemical labels. We therefore defined a different, simpler, and lightweight model for arrow detection. Both of these models are described below.

### Main Object-Detection Model

**Figure 5:**
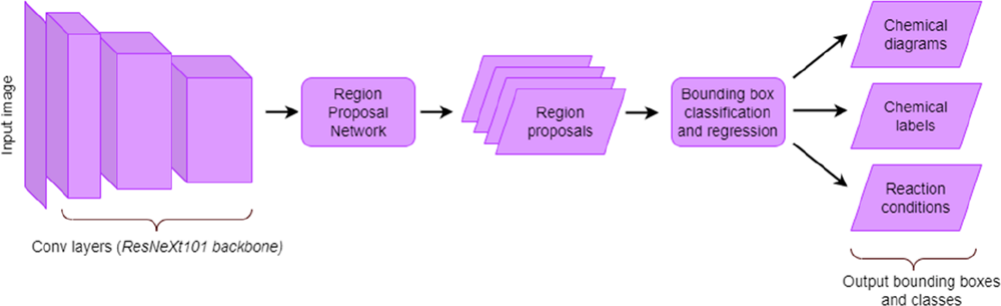
Overview of the main object-detection model. An input image containing the full reaction scheme is fed through a convolutional feature extractor (ResNeXt101), and the output feature maps are then passed to a Region Proposal Network, where regions of interest are selected and regressed. After being passed through fully connected layers, the learned class-specific information is used to classify and further regress bounding boxes to output image patches containing chemical diagrams, chemical labels, and arrow annotations.

We use an object-detection model from the Detectron2^42^ library. The model is based on a Faster R-CNN architecture with a ResNeXt-101^43^ feature-extraction backbone, and a simplified overview has been sketched in Figure 5. We used 2000 synthetically generated reaction schemes to train the object-detection model over 5000 iterations via the means of transfer learning using an available pretrained model as a starting point. We also experimented with a larger training set, but no significant improvement was found, likely due to the small set of unique annotated data used to create the final training sets. We used distance intersection-overunion (DIoU) loss for bounding box regression with relative weights of 2.0 and 10.0 for the region proposal network and the main detection head, respectively; and the default weights for the classification heads. We optimized the neural network using a stochastic gradient descent (SGD) optimizer with a learning rate of 0.001.

### Arrow-Detection Process

**Figure 6:**
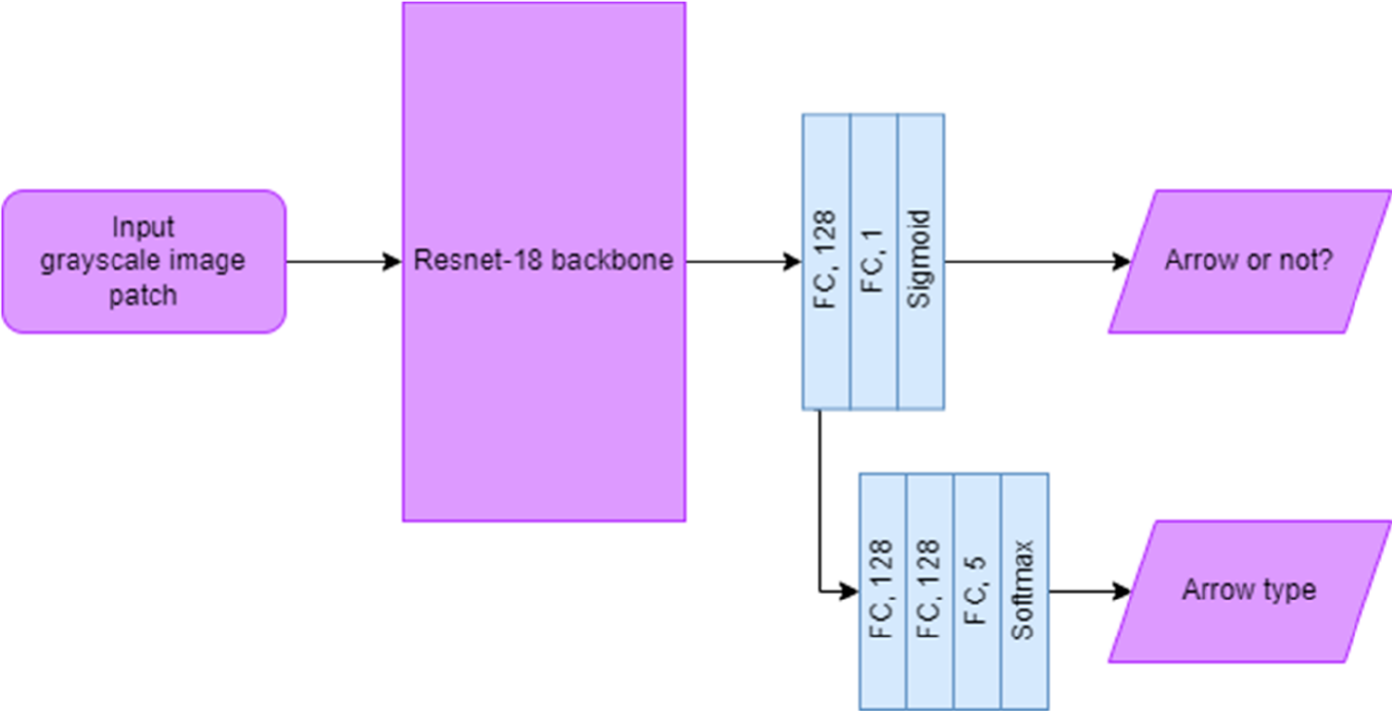
Architecture of the arrow-extraction model. The model takes in an image patch which contains a single, isolated connected component. Features are extracted by the Resnet-18 backbone layers. Transformed features are fed to two parallel branches: one for arrow detection, and another for classification of arrows into arrow types (solid, curly, equilibrium, resonance).

**Figure 7:**
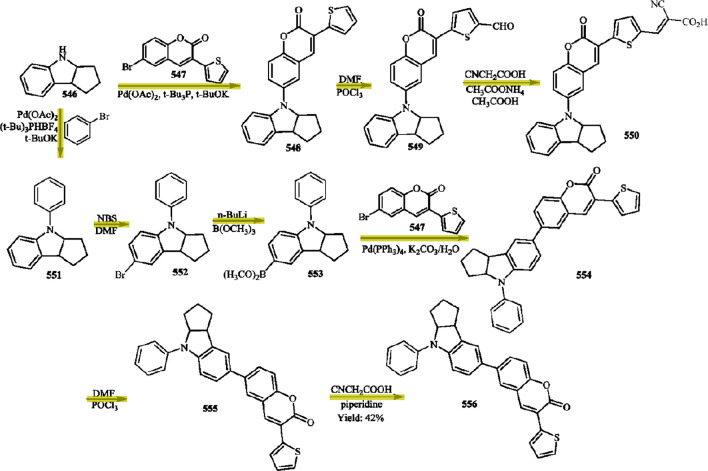
Application of the arrow detection process for the worked example, highlighting the detected solid arrows in yellow (no other arrows were found).

The arrow-detection process is summarized in Figure 6. The arrow-detection model uses a simple convolutional neural network with a Resnet-18^44^ backbone which receives as input a 64 × 64 image patch containing a single connected component and has two branches: the first branch classifies the input patches as those that contain arrows and those that do not. A second branch takes the final features and transforms them further using two additional fully connected layers to further separate the arrows into four classes (solid arrows, curly arrows, equilibrium arrows, and resonance arrows). We use transfer learning from a pretrained Resnet-18 backbone, and train the model end-to-end for 20 epochs using an Adam optimizer with learning rate of 0.001 by applying a binary cross entropy loss to the first (detector) branch and a cross entropy loss on the second (classifier) branch:
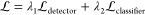
1where λ_1_ = 10 and λ_2_ = 1. To train the model, we used a small number (ca. 150) of annotated chemical reaction schemes, extracted all of their individual connected components, and assigned an arrow classification label from 0 to 5, where 0 represents a nonarrow patch, and classes 1–4 denote the different types of arrows, to each extracted connected component. We added to this training set a small number of arrows that had been created using chemical drawing software and augmented these arrows using affine transformations and by randomly applying a small Gaussian kernel to account for arrows in low-resolution images. This manual addition of specially crafted arrows ensures that there is a greater diversity of arrows in the training set, which affords a more balanced data set. The detected arrows for the worked example are highlighted in Figure 7.

### Diagram Postprocessing

**Figure 8:**
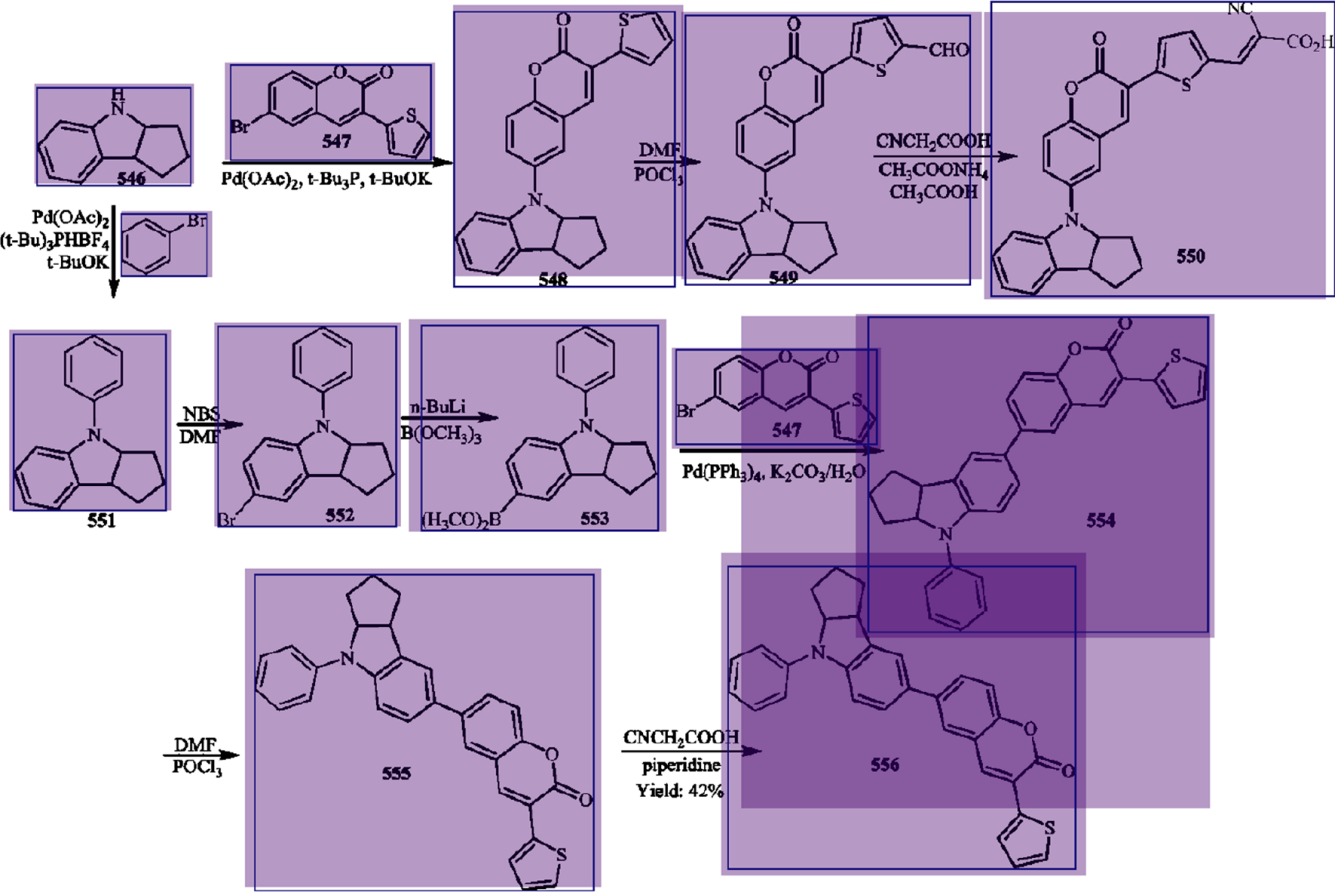
Initial diagram-detection result and the diagram postprocessing step of the operational pipeline. Raw detections (purple regions) are postprocessed to give final predictions (purple outlines).

It is important that the bounding boxes of the relevant image information contain the exact regions of interest in achieving the best OCSR accuracy. In generic object-detection applications, images contain more information than chemical reaction schemes, whose backgrounds are usually noninformative and whose chemical diagrams contain signal discontinuities (e.g., where a superatom is present). Consequentially, our detection process could erroneously identify a chemical diagram because it misjudges its true size. On the one hand, it could afford an incomplete chemical diagram because it missed some of its relevant features in an information-poor region of the image in which the full diagram is contained. On the other hand, it may capture irrelevant information in addition to the chemical diagram because it struggles to partition the contents of this diagram from its surrounding environment. To resolve these inconsistencies, we apply a similar dilation algorithm to that reported for ReactionDataExtractor v1.0; i.e., for each detected diagram, we take the largest detected connected component and dilate it according to a locally calculated dilation kernel, and then assign all individual connected components to that diagram. The effect of this regularization procedure within the diagram postprocessing is shown in Figure 8. It can be seen that the process helps to reduce the number of spurious detections and additionally fine-tunes the boundaries of the detection bounding box to include terminal superatoms.

### Text-Element Postprocessing: Identifying Chemical Labels and Arrow Annotation Information

**Figure 9:**
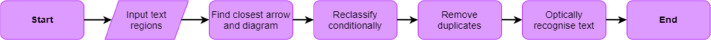
Text-region postprocessing workflow.

From an object-detection point of view, text elements that contain chemical labels and arrow annotations share a high level of similarity. They comprise blocks of text characters that are challenging to differentiate without optically recognizing and parsing the text, the latter being outside the scope of computer vision. An important visual cue for such a text, which can be utilized by an object detector, is its relative position within an image. The positions of arrow annotations and their corresponding reaction-step arrows are correlated, as is the position of a chemical label and its corresponding chemical diagram. As expected, we found that training the object-detection model with two text-element classes instead of a single class achieved higher performance and that the model did not require the explicit use of an attention mechanism. Nevertheless, as mentioned earlier, only a small number of unique training data were available due to the high labor cost of data annotation, which is why a postprocessing step was also incorporated into the operational pipeline of ReactionDataExtractor v2.0. This step enforces the aforementioned inductive biases. Figure 9 summarizes the full set of operations in this text-element postprocessing step.

**Figure 10:**
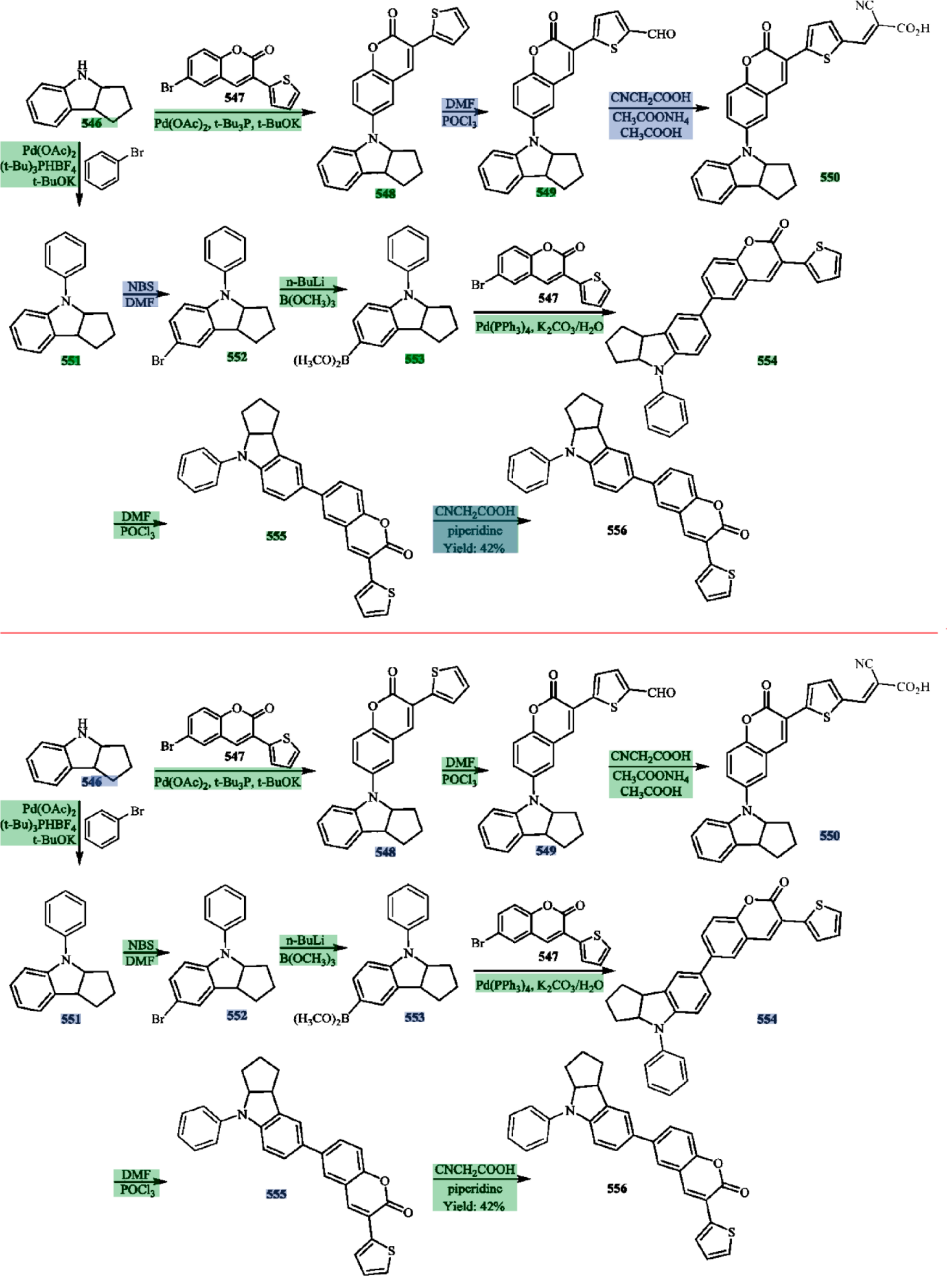
Application of the text-element detection and postprocessing steps of ReactionDataExtractor to the worked example. The two panels depict detected chemical labels and arrow annotations before (top) and after (bottom) the postprocessing step. Regions labeled as arrow annotations are marked in green, while chemical labels are colored in blue. After text-element postprocessing, the raw predictions from the text-element detection step are conditionally reclassified based on simple heuristics. The text-element postprocessing step mainly improves the performance of detecting arrow annotations, but it also helps when the model prediction with different class labels overlap (bottom part of the top panel).

**Figure 11:**
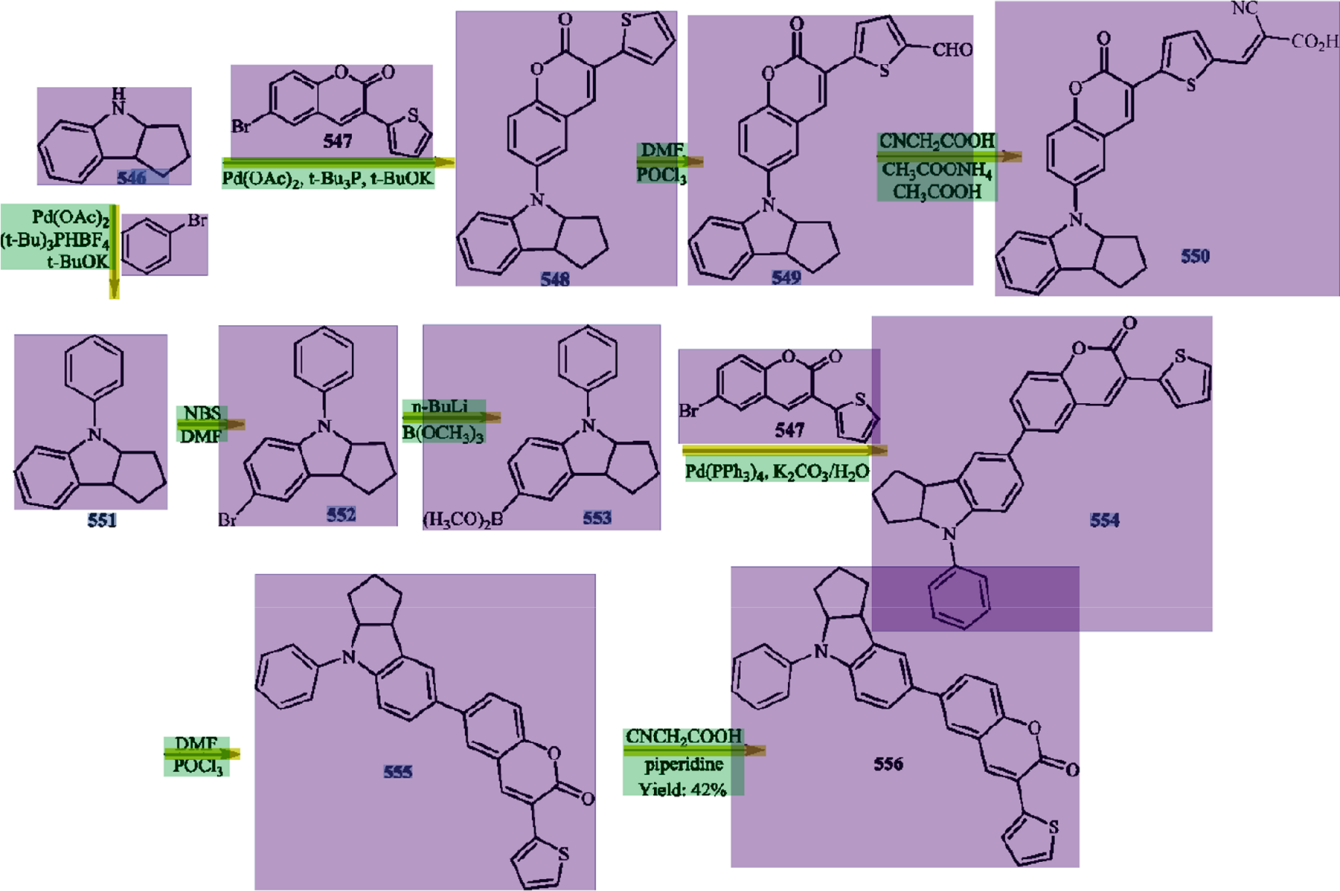
Final predictions for chemical diagrams (purple), arrows (yellow), arrow annotations (green), and chemical labels (blue). Note that not all labels are mutually exclusive, for example a region can contain a chemical diagram which belongs to a contained set of arrow annotations.

For each text-element region detected by the neural network either as a chemical label or as a contained set of arrow annotations, we find the closest chemical diagram and reaction arrow. We update the prior class to chemical label if the closer of the two is a chemical diagram and the text-element region of interest is below this diagram. If, however, a reaction arrow is the closer of the two and this region satisfies a directionality criterion with respect to this arrow, then the prior class is changed to a set of arrow annotations. The directionality criterion checks whether the text-element of interest lies along a line normal to, and passing through, the center of this arrow; for example, in the case of a horizontal arrow, this criterion checks whether this region lies either below or above this arrow within a certain distance from this arrow. Otherwise, we kept the prior class for this region of interest. Then we pair each label with its nearest diagram and each region containing a set of arrow annotations with its nearest arrow. We show the importance of this step in Figure 10. In particular, the textual regions that describe a contained set of arrow annotations, which are characterized by a larger semantic diversity, suffer from having been trained using few unique data. We quantify the importance of this step by performing an ablation study, as described later in the technical evaluation section. The performance of this step can be further improved with more data and incorporation of additional scheme patterns through user-defined schema. Nevertheless, the text-element postprocessing step improves the text-identification performance of ReactionDataExtractor. Prior to reconstructing the chemical reaction scheme, we assigned relevant chemical diagrams to regions of arrow annotations. The final output is shown in Figure 11.

Finally, we performed OCSR using the DECIMER^45^ package. We used the Tesseract^46^ software to decode the text elements of the images, which have been classified as chemical labels or arrow annotations.

### Graph Formation

A graph of the full chemical reaction scheme was then constructed by applying a search algorithm around each arrow in the images. We estimated the direction of a given arrow by fitting a minimal rotated bounding rectangle to its contour using a least-squares fit. We then calculated the center-of-mass (COM) coordinates of this arrow from the constituent pixels. The COM is used to decide which side of an arrow represents the chemical reactants and products of a given step. This approach does not work for curly arrows, which are more complex, and a rotated bounding rectangle does not properly capture directionality. Typically, curly arrows have up to 4 end points (2 arrow hooks, and 2 ends), and these end points need to be captured in order to fully understand the local environment around the arrow. To do this, we undertake the following heuristics:

We first look at the boundaries of the original (unrotated) arrow bounding box. We select four rectangular regions, one per each side of the bounding box. One side of each rectangle is defined by the width or height of the bounding box, whereas the other side is equal to a fraction of the other dimension, so that the regions near the boundaries of the bounding box can be probed. These regions usually contain all arrow end points. Of all arrow pixels, we select one pixel per rectangle such that its coordinates are closest to the probed boundary (for example, in the region close to the left boundary, we select the leftmost arrow pixel). We then filter these pixels in two stages. In the first stage, we filter pixels that are very close to each other in one arrow end that spans two regions. In the second stage, we filter pixels that are not end points. This can happen if an arrow is curved outward. In this case, we erase all pixels from a given probed region and check if the arrow is still a single connected component. Removing pixels around an end point causes no effect on the integrity of an arrow, but if pixels around the middle are removed, the arrow breaks into two separate connected components.

Once we have the end points, we find the arrow hooks by fitting straight lines in the regions around the selected pixels using a least-squares fit. We then perform a scanning operation in the direction perpendicular to the fitted line and check whether the number of arrow pixels is approximately constant or varies. In the former case, we classify this end point as an arrow end; otherwise this is deemed to be an arrow hook.

Finally, we select one arrow hook and one arrow end by checking the size of chemical diagrams closest to them and choose the hook and end with the largest diagrams nearby. Here we assume that in each step there is a main reactant and product, as well as potentially side reactants and products, depictions of which are strictly smaller than the image size of the main chemicals displayed in the chemical reaction.

In order to scan around the arrow, we compute two separate directions, one for a scan of main reactants and one for a scan of main products, from slopes of lines connecting the midpoint of the arrow bounding box and the selected arrow end and hook, respectively. This generalizes the algorithm, which was originally devised for ReactionDataExtractor v1.0, such that it can now process different kinds of reaction arrows (including resonance, curly, and equilibrium arrows). While for a complicated sequence of reactions, classifications for the role of chemicals in a reaction, such as ’reactants’ and ’products’, might not be useful, e.g., for catalytic cycles, they are well-defined for each individual reaction step. We thus perform two scans to find “reactants” and “products” of a step by marking equidistant lines along the derived direction as one moves away from the center of an arrow. We stop the scan when another arrow is encountered (representing a different reaction step) and check for bounding boxes found in each scanning step. If no “products” or “reactants” are found, it is assumed that the individual reaction step concerned is spread over multiple lines; therefore, a search is then performed in the previous or next line, depending on the position of an arrow relative to the image size. Individual reaction steps are then connected into a graph structure by matching “products” of one step with “reactants” of another. The graph can then be exported to an easily processable format. The full reaction graph for the worked example is depicted in Figure S3.

## Technical Evaluation

### Overall Pipeline Performance

The performance of our pipeline was formally measured using precision, recall, and *F*-score metrics, which are defined as
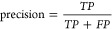
2
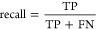
3
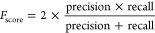
4where TP (true positives) denote correctly extracted data, FP (false positives) symbolize incorrect extracted data, and FN (false negatives) refer to the data that were not detected by our system. Further details on the metrics that were used in the context of evaluating the accuracy of individual image-processing steps are provided in the Supporting Information.

Each step of the operational pipeline of ReactionDataExtractor was evaluated wherever it was new or it has been altered since the production of ReactionDataExtractor v1.0, i.e., the steps involving the extraction of chemical diagrams, arrows, arrow annotations, and chemical labels, as well as matching between chemical labels and their corresponding diagrams, and the reconstruction of the overall chemical reaction scheme. In contrast to the evaluation exercise for ReactionDataExtractor v1.0, we herein limit evaluation of extracting arrow annotations to an assessment of whether correct regions were detected, and we do not consider explicitly if the chemical species that were extracted are correct. This is for two reasons: (a) the engine used for optical character recognition in ReactionDataExtractor v1.0 remained the same for v2.0; (b) a large, diverse test set was created to evaluate the ReactionDataExtractor pipeline, which was enabled thanks to the reduced cost of annotation compared to manual text annotation that was required for v1.0. In common with the evaluation process for ReactionDataExtractor version 1.0, version 2.0 was assessed using figures from open-access articles across a wide variety of scientific journals from two mainstream publishers: Springer and the Royal Society of Chemistry. A notable difference between these evaluation processes is that the process for assessing v2.0 was much less constrained as there are currently no scope limitations, which were natural when rule-based techniques were applied for ReactionDataExtractor v1.0. The scraping process is described in-depth below.

Figures from articles published in Springer journals were scraped by using the API that was provided by the publisher. A search was performed using keywords “reaction” and “scheme” to make no assumptions about the layout of the reaction schemes or the domain of chemistry from which they originate. The first 1542 articles were downloaded in HTML format and analyzed to identify images of reaction schemes using their names. Thereby, schemes were selected using a pattern-matching procedure, and the first 360 of these schemes were selected. Using these names, we downloaded the 360 schemes from the publisher’s servers.

Similarly, reaction schemes from articles published in journals across the Royal Society of Chemistry publication portfolio were scraped using the advanced user search facility. A similar search was performed using the same keywords: “reaction” and “scheme”. 1000 articles were downloaded in HTML format and analyzed for images using their names and pattern matching; a procedure similar to the one above. The first 200 schemes were selected, and download was attempted from the publisher’s servers. Of these, 43 downloads raised a HTTP exception during the process and were discarded. This process therefore yielded 157 reaction schemes.

In contrast to ReactionDataExtractor v1.0, we do not make any assumptions about the reaction schemes in v2.0. Naturally, there are still boundaries that define a chemical reaction scheme (e.g., some species should be presented as chemical diagrams in order to be captured); yet, in the vast majority of cases where these boundaries are violated, the pipeline will raise an exception and skip an image automatically.

Table 1 summarizes the precision, recall, and *F*-score metrics for the relevant steps of the operational pipeline in ReactionDataExtractor v2.0. Over 1100 arrows and 2500 chemical diagrams were present in the 517 reaction schemes that comprised the evaluation test set.

**Table 1:** Evaluation Metrics of the Key Steps in the Operational Pipeline of ReactionDataExtractor v2.0^a^

pipeline deliverable	TP	FN	FP	recall	precision	*F*-score
arrow detection	1131	43	51	96.3%	95.7%	96.0%
diagram detection	2292	237	84	90.6%	96.5%	93.5%
label detection	1439	464	504	75.6%	74.1%	74.8%
arrow annotation detection	756	299	167	71.7%	81.9%	76.4%
diagram-label matching	1255	235	N/A	84.2%	N/A	N/A
overall reaction graph evaluation	878	289	120	75.2%	88.0%	81.1%

a For diagram-label matching, we assessed correct and incorrect matching, denoted them as true positives and false negatives, respectively, and computed the equivalent recall metric.

**Figure 12:**
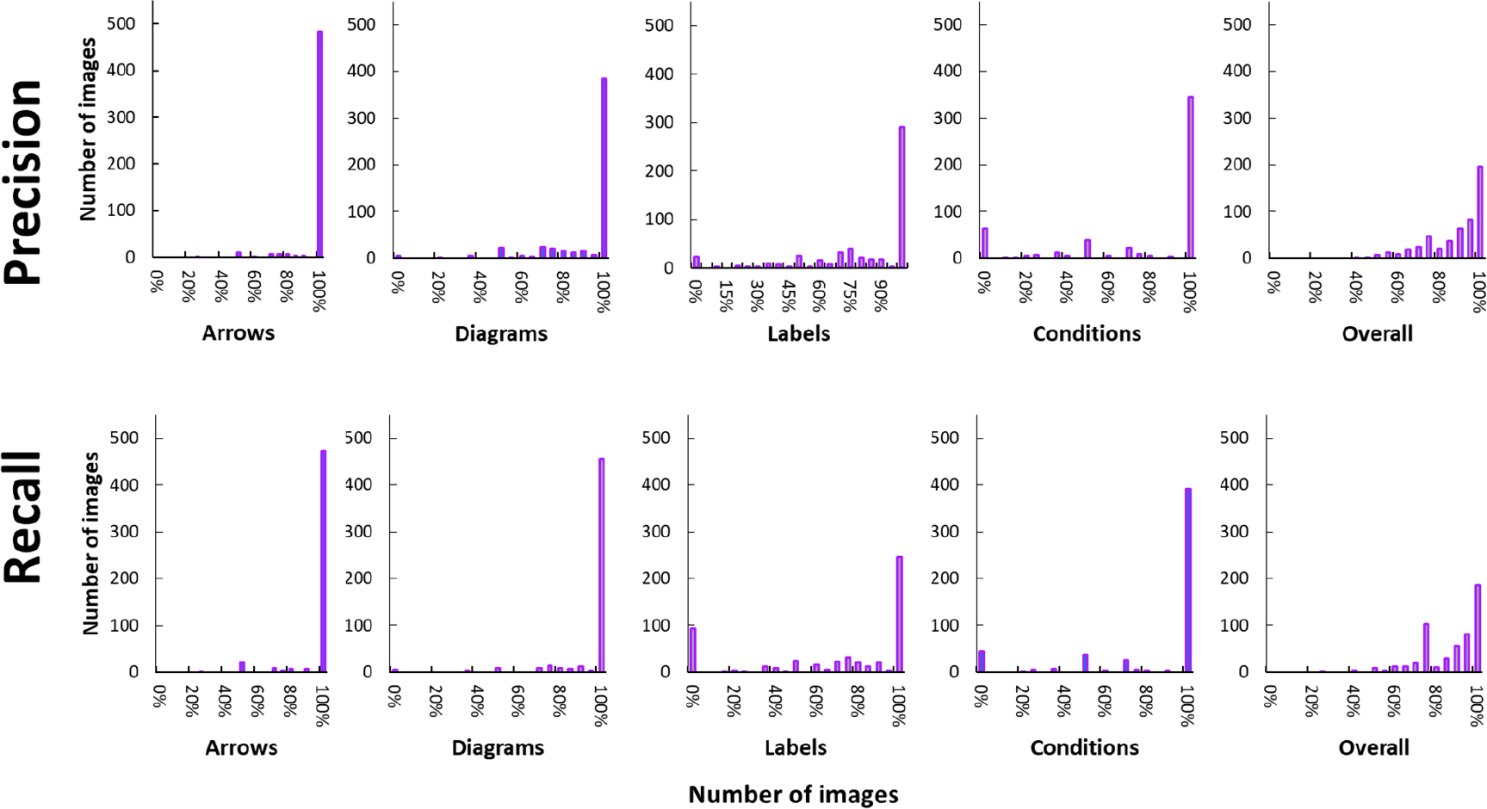
Distributions of precision and recall metrics for pipeline evaluation across the entire evaluation data set.

*Representative Performance Metrics*. We computed distributions of precision and recall values for the same four stages as well as their average in individual images; these are provided in Figure 12. These distributions indicate that the pipeline works well across the whole data set. The only exceptions are the precision and recall distributions for label detection, where a peak is clearly visible at 0%. This is likely due to cases where labels are of a particularly small size, since these are more challenging for the main detection model to extract. The distributions for the overall detections show that objects in almost 200 schemes have been detected with perfect precision or recall (196 and 186 reaction schemes, respectively).

The resulting metrics and sources of error are further discussed in what follows. Qualitative examples referred to in the text below are presented in Supporting Information.

The arrow-detection stage afforded a precision of 95.7% and a recall of 96.3%. This is excellent performance with only a very small fraction of arrows that have not been correctly detected. Some curly arrows, in particular, present a higher complexity that requires high model generalization performance. Moreover, some arrows cannot be captured by the model since they do not constitute isolated connected components and are glued to other elements of a reaction scheme.

The detection of chemical diagrams achieved an overall precision of 96.5% and a recall of 90.6%. Given a simple definition of a chemical diagram (all chemical species with at least one single bond line are considered to be chemical diagrams), these metrics suggest strong performance. Incorrect detections are mainly due to poor diagram postprocessing, whereby the dilation process is not sufficient to cover distant superatom connected components (cf. Figures S4, S5).

Compared with the detection of arrows and chemical diagrams, the performance of ReactionDataExtractor v2.0 is lower in detecting textual elements that pertain to chemical labels and arrow annotations. Apart from reasons specific to these two classes of information, the detection process struggles to differentiate between relevant text regions (the two classes) and other text (such as headers, footers, and auxiliary information). Such differentiation relies primarily on contextual information (the local environment of each image region), which is affected by the density of features (crowding) within an image. Additionally, the relevant semantic features of data belonging to textual element classes are challenging to model fully using a sparse data set.

The detection of chemical labels led to a precision metric of 74.1% and a recall of 75.6%. Label detection is compromised, particularly where elements of a reaction scheme cannot be found in the set of data that trained the detection model. For example, in Figure S4, a dashed horizontal line appears to derail label detection. The detection also seems to be negatively affected in reaction schemes that describe synthetic details from the domain of organometallic chemistry (Figures S6 and S7). The reason here is likely to be the same issue: insufficient relevant data within the training set. While ReactionDataExtractor has been designed with organic-chemistry applications in mind, readers with an interest in organometallic chemistry could possibly improve the label-detection performance by retraining the detection model with more organometallic compounds in the training set.

The detection of text from arrow annotations results in precision and recall values of 81.9% and 71.7%, respectively. One particular issue is the presence of other, nonimportant, crowded text (Figure S8) . Additionally, these image regions manifest with much greater semantic complexity and variety in scale compared to chemical labels, and therefore they require a much larger number of training samples to more fully model the class of text. This is particularly visible in certain figures (e.g., Figure S9 and S10), where parts of chemical diagrams are erroneously marked as arrow annotations.

Finally, we also performed an overall evaluation of the entire operational pipeline of ReactionDataExtractor v. 2.0 using the output reaction graphs as a proxy method. Indeed, it is challenging to provide a single metric for the whole pipeline. So, this workflow captures all the crucial elements that form each reaction scheme graph; the workflow is equally sensitive to possible issues with the overall reaction graph reconstruction.

Most reaction schemes are composed of a series of ordered reaction steps. In order to probe the graphs, the algorithm constructs a reaction graph from the annotated data by marking and numbering individual reaction steps and assigning diagrams to these reaction steps. It then finds starting nodes both in the annotated data and pipeline output and traverses the graphs, starting from the initial node (or multiple initial nodes) and storing individual reaction steps. As it traverses, it compares the reaction steps from annotation data and pipeline output and counts the number of matched reaction steps (true positives), reaction steps absent in the output (false negatives), and spurious reaction steps (false positives). It handles special cases of cyclic reaction schemes, where no starting nodes are found, by selecting a random node as the starting node, forming a circular traversal path ending with the same starting node and comparing the reaction steps along the way. The details of the matching process are provided in the SI. This evaluation process achieved an F-score of 81.0%, which indicated good performance. This evaluation relies on correct chemical diagram detections (used for matching the steps) as well as correctly detecting the arrow and reconstructing reaction steps and is very sensitive to incorrect predictions, thereby giving a good metric for assessing the overall performance of ReactionDataExtractor v.2.0.

Overall, very good performance metrics are achieved, given the small data sets that were used to train the main object-detection and arrow-detection models. Nevertheless, it is clear that certain stages of the operational pipeline for the tool could benefit from expanding the size of the data set used in training its various models. This is especially the case for the detection of chemical arrow annotations. It could also be helpful to introduce a more varied schema for the synthetic generation of artificial reaction schemes.

### Assessing the Importance of a Separate Arrow Detection Model

Our arrow detection model is an entity that is separated from the main object detection model. The detection of the reaction arrows should be simple: they contain few visual features and are easily distinguishable from other detected objects. It is therefore important to justify the decision to separate the detection of arrows from the main object detection model. To achieve this, we performed an ablation study of the main detection model, whereby we added a fourth label to detect reaction arrows and trained the model using the same artificial reaction schemes with additionally annotated arrow bounding boxes. The results are shown in Table 2. In this test, we evaluated only the detection part of our developed classifier, in common with our main evaluation.

**Table 2:** Ablation Study^a^

		TP	FN	FP	recall	precision	*F*-score
arrow detection	baseline	1131	43	51	96.3%	95.7%	96.0%
	single model	903	326	129	73.5%	87.5%	79.9%
diagram detection	baseline	2292	237	84	90.6%	96.5%	93.5%
	single model	1996	99	204	95.3%	90.7%	92.9%
label detection	baseline	1439	464	504	75.6%	74.1%	74.8%
	single model	1319	545	485	70.8%	73.1%	71.91%
arrow annotation detection	baseline	756	299	167	71.7%	81.9%	76.4%
	single model	491	333	384	59.6%	56.1%	57.8%

a The ablation study was used to investigate the effect of incorporating the arrow detection model into the main object detection model as an additional label. Baseline is our current workflow with a main object detection model and a separate arrow detector, whereas the single model scenario is where the main object detection model is trained to additionally detect reaction arrows.

From the results of this experiment, it can be inferred that arrow detection suffers from incorporating the task into a single model. The *F*-score of 79.9% is very good but can be significantly improved by delegating the detection exercise to a simpler classifier. One particular limitation of the classifier is that the connected components have to be well-separated for it to work well, as opposed to the main object detection model, which has no such limitation. This can be problematic, when individual letters within arrow annotation overlap with an arrow, but such issues are present in our evaluation data set and the model still compares favorably with the object detection model. One consequence of the higher performance is a higher metric for arrow annotation detection, which benefits from true positive arrow detections, as this information is used during the text-element postprocessing stage.

### Assessment of the Combined Arrow Detection/Classification Model

Our main evaluation shows the results of the arrow extraction process without specifying the detected arrow type. To provide a full picture, we provide this breakdown in Table 3.

**Table 3:** Full Breakdown of the Combined Arrow Detection/Classification Model Metrics on Our Evaluation Set^a^

	TP	FN	FP	recall	precision	*F*-score
arrow detection	1131	43	51	96.3%	95.7%	96.0%
solid A. classification	1071	48	38	95.7%	96.6%	96.1%
curly A. classification	33	5	17	86.8%	66.0%	75.0%
equilibrium A. classification	12	4	5	75.0%	70.6%	72.7%
resonance A. classification	0	0	6	N/A	0%	N/A

a In the first row, the overall metrics for arrow detection are shown (c.f. Table 1), and in rows 2–5, evaluation of the classification task is shown. This task is assessed independently from the detection task—detected arrows are included in false negatives in rows 2–5. In the evaluation set, no resonance arrows were present.

Our test set contained no resonance arrows. The table shows that recall values are 75% or above across all remaining classes and high values for precision. We note that the model takes as input *all* connected components in all images. Given this information, the number of false positives across all classes can be considered to be low.

### Assessing the Importance of the Text Postprocessing Routine

We have assessed the importance of the bias injection performed in the text postprocessing routine on the overall performance of the pipeline in text element detection. We report below metrics on the original evaluation set for arrow annotations, and chemical labels before and after the postprocessing step (Table 4).

**Table 4:** Ablation Study Assessing the Importance of Text Element Postprocessing

		TP	FN	FP	Recall	Precision	*F*-score
labels	raw	1254	649	454	65.9%	73.4%	69.4%
	postprocessed	1439	464	504	75.6%	74.1%	74.8%
arrow annotations	raw	698	359	439	66.0%	61.4%	63.6%
	postprocessed	756	299	167	71.7%	81.9%	76.4%

From the comparison, it is notable that while most metrics remain the same, the number of false positives is drastically reduced in the arrow annotations class, leading to a significant increase in the precision value (from 61.4% to 81.9%). Hence, the postprocessing method limits the number of spurious detections.

### Comparison with ReactionDataExtractor v. 1.0

We quantitatively compared ReactionDataExtractor v.2.0 to its predecessor. It is important to note the limited scope of operation for ReactionDataExtractor v.1.0., which was found to be capable of extracting ca. 30% of reaction schemes that one can find in the academic literature, as reported in the original publication.^37^ This is because ReactionDataExtractor v.1.0 carries the intrinsic limit that it can process only simple depictions of reaction schemes. Nevertheless, a fair comparison can be made by evaluating our current pipeline on the v.1.0 evaluation data set. We compared the detection of reaction arrows, chemical diagrams, and labels, as data extraction of arrow annotations is not directly comparable due to differences in the methodology used (in v.1.0, this part of the data extraction included optical character recognition). The results have been collected in Table 5.

**Table 5:** Comparison between ReactionDataExtractor v. 1.0 and our current pipeline (v. 2.0)

	version no.	TP	FN	FP	Recall	Precision	*F*-score
arrow detection	1.0	370	48	59	88.5%	86.2%	87.4%
	2.0	407	11	56	97.2%	89.9%	93.4%
diagram detection	1.0	722	149	103	82.9%	87.5%	85.1%
	2.0	783	88	51	89.9%	93.9%	91.9%
label detection	1.0	375	79	143	82.6%	72.4%	77.2%
	2.0	379	74	134	83.6%	73.9%	78.4%

Our current pipeline outperforms its predecessor in all areas, as documented by the reported *F*-scores, confirming superiority of the neural network model over detection using hand-crafted features. The very high precision of arrow detection for ReactionDataExtractor v2.0 is also noteworthy. In this evaluation set, only solid arrows were present and these are particularly easy to detect using our current arrow detection model.

## Conclusions

ReactionDataExtractor v2.0 is a cheminformatics tool that has been designed for automatic extraction of chemical reaction schemes and conversion of them into database-ready formats. The tool detects all salient elements of a reaction scheme using approaches that are based primarily on deep-learning algorithms. The tool also links its textual elements (chemical labels and arrow annotations) to their parent regions (arrows and chemical diagrams) via rule-based approaches. Once all relevant chemical information was extracted from a reaction scheme, the tool reconstructs the context of the entire reaction from its individual elements to produce it in a standardized format. These approaches also serve as a way of injecting expert knowledge into the pipeline on a path to make it “chemistry-aware”. ReactionDataExtractor v2.0 further integrates state-of-the-art optical character and chemical structure recognition engines to afford an independent chemical reaction scheme extraction tool.

Compared to ReactionDataExtractor v1.0, ReactionDataExtractor v2.0 is considerably simpler, as its extraction process is performed primarily by two deep-learning architectures–a simple convolutional classifier for arrow detection and a two-step object-detection model that detects the remaining regions of interest within the analyzed image. This approach simplifies the operational pipeline of the tool, while retaining modularity and enabling potential for further improvement, as the models can be retrained with addition of alternative data that may suit the specialized needs of certain domains of synthetic chemistry; or object-detection models could be swapped for more contemporary object detectors as the technology advances. Several postprocessing stages were introduced to further improve the raw results, and this resulted in a significant improvement in detection, especially with regards to the textual region classification.

We evaluated the operational pipeline for ReactionDataExtractor v2.0, using a large set of images from open-source scientific journal articles. The scope of its pipeline is not constrained by the particulars of the reaction scheme as long as chemical diagrams and arrows are present. Hence, the tool requires no associated selection process, which was an important obstacle for generating databases using ReactionDataExtractor v1.0. The test set used to evaluate ReactionDataExtractor v2.0 is available at www.reactiondataextractor.org. Current sources of errors have been discussed, with qualitative examples provided in the Supporting Information.

The models in ReactionDataExtractor v2.0 were trained using synthetic data that were generated from a collection of image patches that had been scraped from chemical databases and supplied in the form of a small number of annotated images. These synthetic data were generated using our new Scheme Engineer module within ReactionDataExtractor v2.0, which ensures that inductive biases are injected into the image-based identification of reaction schemes. The integration of the synthetic data generation capabilities of Scheme Engineer within the new operational pipeline of ReactionDataExtractor v2.0 will facilitate the training of its built-in deep-learning models for object detection. Combining such functionalities within ReactionDataExtractor allows its extensibility and provides a further scope for further improvement of the tool.

While the model significantly expands on the capabilities of ReactionDataExtractor v1.0, it comes with limitations. The Scheme Engineer, as a method based on data augmentation, suffers from poor out-of-distribution generalization and requires one to define new scheme patterns whenever schemes of interest do not align with the modeled data distribution. The models could be further improved with more data, requiring potentially expensive data annotation. Finally, we note the lack of a single object detection model in the current release, as we decided to split arrow detection into a separate model. The goal of combining these models into a single model, without compromising the metrics, is a subject of potential future work.

## Data Availability

All the source code in this publication is freely available as an open-source package under an MIT license at http://www.reactiondataextractor.org/downloads and https://github.com/dmw51/reactiondataextractor2. ReactionDataExtractor uses **DECIMER**, an open-source package (MIT license) that is freely available at https://github.com/Kohulan/DECIMER-Image_Transformer. The data set, produced by ReactionDataExtractor as part of the evaluation, and its annotations, and the evaluation files are available at http://www.reactiondataextractor.org/evaluation. A list of all data is given in the Supporting Information. The associated web scraping code that was used to obtain the open-access articles is available at http://www.reactiondataextractor.org/evaluation. An interactive online demo of ReactionDataExtractor is available at http://www.reactiondataextractor.org/demo, and a user guide is available at http://www.reactiondataextractor.org/docs.

## Supporting Information Available

Strict definitions of the evaluation metrics at each stage, a list of figures used in the evaluation set, details on the overall evaluation process and information on how to reproduce the evaluation of this work (PDF)
