# Supplementary material for: ReactionDataExtractor 2.0: A Deep Learning Approach for Data Extraction from Chemical Reaction Schemes
Source: J Chem Inf Model. 2023 Sep 20;63(19):6053–67. doi: 10.1021/acs.jcim.3c00422 (PMC10565829; doi:10.1021/acs.jcim.3c00422)
Supplement: Supplementary file 1 — ci3c00422_si_001.pdf [file ci3c00422_si_001.pdf]

Supporting Information for:

ReactionDataExtractor 2.0: A deep learning approach for data extraction from chemical reaction schemes

Damian M. Wilary,[1] Jacqueline M. Cole[1,2,*]

[1] Cavendish Laboratory, Department of Physics, University of Cambridge, J. J. Thomson Avenue, Cambridge, CB3 0HE, UK

[2] ISIS Neutron and Muon Source, STFC Rutherford Appleton Laboratory, Harwell Science and Innovation Campus, Didcot, Oxfordshire, OX11 0QX, UK

Corresponding Author: Jacqueline M. Cole. Email: *jmc61@cam.ac.uk*

Contents

Metric definitions for evaluation 2
Graph Traversal – overall evaluation 3
How to reproduce the evaluation 6
Reconstruction graph 6
Image evaluation – challenges 8
High level comparison between version 2.0 and 1.0 12
Details of the main object detection model 13

Metric definitions for evaluation

All sections of the pipeline are evaluated independently, even if a section relies on correct output from a previous step (i.e. the error is propagated).

Arrow detection

True positive: where a panel corresponding to an arrow was correctly classified as an arrow. Measured using intersection over union (IoU) with a threshold of 0.5.

False positive: where a panel not corresponding to an arrow was incorrectly classified as an arrow (No true arrow region has IoU above 0.5 with the marked region).

False negative: where true panel corresponding to an arrow was not found (either not marked as a proposal, or incorrectly classified; No marked arrow has IoU above 0.5 with the true diagram).

Conditions detection

True positive: where a panel corresponding to a reaction conditions region was correctly marked. Measured by IoU with a threshold of 0.5.

False positive: where a panel spuriously marked as a reaction conditions region is found (No true conditions region has IoU above 0.5 with the marked region).

False negative: where true reaction conditions panel has been omitted by the model (No marked conditions region has IoU above 0.5 with the true region).

Diagram detection

True positive: where a panel corresponding to a chemical diagram was correctly marked. Measured by IoU with a threshold of 0.5.

False positive: where a panel spuriously marked as a chemical diagram is found (No true diagram has IoU above 0.5 with the marked diagram).
False negative: where true chemical diagram panel has been omitted by the algorithm (No marked diagram has IoU above 0.5 with the true diagram).

Label detection

True positive: where a panel corresponding to a chemical label region was correctly marked. Measured by IoU with a threshold of 0.5.

False positive: where a panel spuriously marked as a chemical label region is found (No true chemical label region has IoU above 0.5 with the marked region).

False negative: where true chemical label panel has been omitted by the model (No marked chemical label has IoU above 0.5 with the true region).

Diagram-label matching

True positive: Where a label was assigned to a correct chemical diagram
False negative: Where a label was assigned to an incorrect chemical diagram

Graph Traversal – overall evaluation

In order to evaluate the overall reconstruction, we traverse both the annotated and output reaction graphs on a reaction step-by-step basis. To achieve this, we first annotate reaction steps in the images and number them (starting from 0, Figure S1), and process the annotations to recover all chemical diagrams in each reaction step. Similarly, we take the output reaction graph, find all starting nodes, and all paths using a breadth-first search. These paths are defined in the final reaction reconstruction step, which is dictated by the found reaction arrows and their directions. We then process these paths to recover all individual reaction steps, number them, and extract all chemical diagrams in each reaction step (Figure S2).

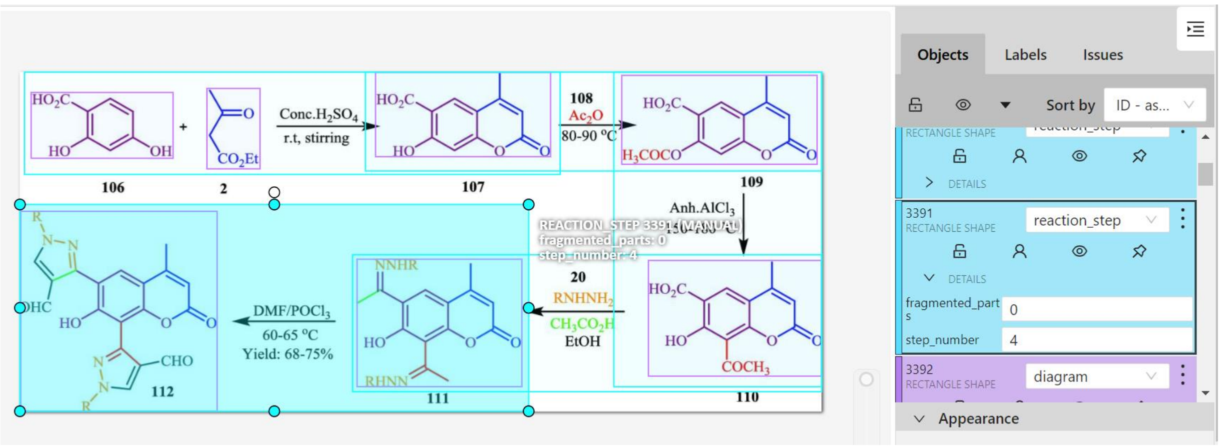

Figure S1: Annotated reaction steps, the highlighted step is the 5th step (index 4 with 0-based indexing), it contains one reactant chemical diagram, and one product chemical diagram. The matching is successful only if an equivalent 5th reaction step is both also found in the output graph and it contains the same chemical diagrams. This relies on a successful reconstruction of initial 4 steps and implicitly enforces which diagram represents a reactant, and which the product of this step, since the reactant of this step has to be a product of the previous step, otherwise the reconstruction would not have been successful.

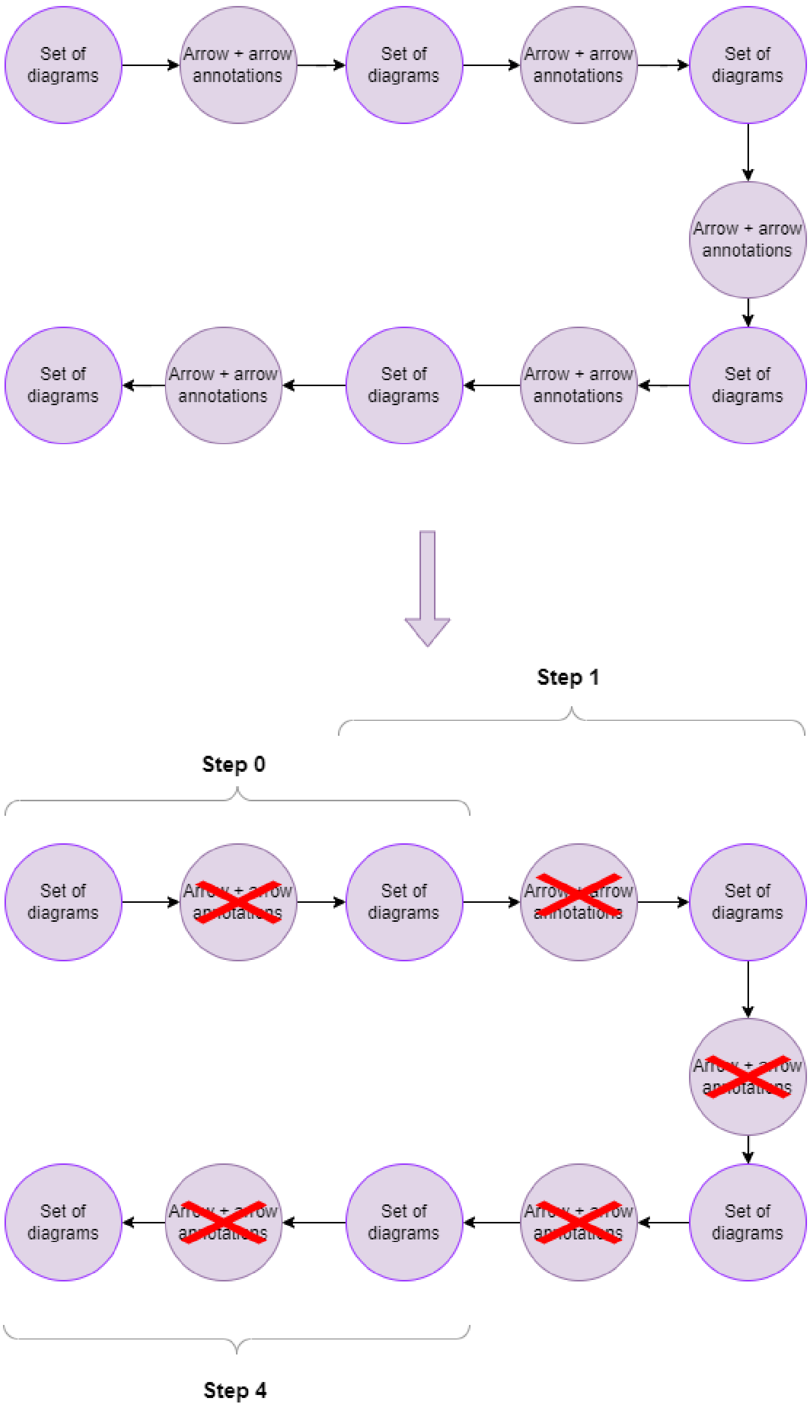

Figure S2: A pictorial representation of the reaction graph reconstructed by the pipeline and the steps required to perform an evaluation. The output is a directed graph of alternating nodes representing chemical diagrams, and arrows and their annotations. The directionality of the graph is dictated by direction of reaction arrows. To process the reaction graph in preparation for overall evaluation, we find the path from start to end, and take every 2nd node to keep only chemical-diagram nodes. We then combine the nodes into reaction steps, and number them to get a representation that is equivalent to that in the annotation file.

We then match the reaction steps from the annotations and output files both using the step number and chemical diagrams in each step. Whereas the step number has to match exactly, we require a mean IoU between all diagrams in a given step of 0.7 – a high threshold that is

very restrictive as to the number of matched diagrams (each false positive or false negative chemical diagram lowers mean IoU significantly) while also allows slight differences between annotation and output bounding boxes for the diagrams.

The special case of cyclic reaction schemes is handled in the same manner, except that the starting node is chosen at random, and a circular path is formed and compared with a path in the annotated schemes starting from the same point.

We define true positives, false negatives and false positives in the following way:

True positive: Where a reaction step was correctly matched (the step number in the output file is correct, and it contains the same chemical diagrams as its annotated counterpart)
False negative: Where an annotated reaction step was not found in the output (either the reaction step was completely missing, or ordering of the output reaction step was incorrect or it did not contain the correct chemical diagrams)
False positive: Where a spurious reaction step was found (based on the same criteria as above).

How to reproduce the evaluation

Files required to reproduce the main evaluation can be downloaded at
http://www.reactiondataextractor.org/evaluation

The zip archive contains:

- A README file that explains step-by-step the process of evaluation
- The produced output files inside evaluate/eval_16Jul
- The script we used to process the images. This script is the main extraction script with its individual extractor objects exposed to allow more control over which data are saved into json files. This script is stored in evaluate/eval_auto_rde2.py
- The script we used to compare output files and annotations and produce our metrics. This script is stored in evaluate/compare_cvat.py
- The annotation files we used stored in CVAT 1.1 format (https://www.cvat.ai/) inside evaluate/annot_rde2testset17Jul.xml
- The evaluation spreadsheets produced from evaluate/compare_cvat.py with all the metrics from the main evaluation stored in Table 1 in the manuscript, as well as the arrow detection/classification single model evaluation stored in Table 2. These evaluation spreadsheets are evaluate/eval_final_17Jul.ods and evaluate/eval_final_arrows_17Jul.ods

Reconstruction graph

Below we present a reconstruction graph for the worked example in the main article.

```
{
    "adjacency": {
        "0": [1,3],
        "1": [2],
        "3": [4],
        "5": [6],
        "6": [7],
        "8": [9],
        "9": [10],
        "4": [11],
        "11": [5],
        "7": [12],
        "12": [13],
        "14": [15],
        "15": [16],
        "13": [17],
        "17": [14],
        "2": [18],
        "18": [8]
    },
    "nodes": [
        [{"smiles": "C1CC2C(C1)NC3=CC=CC=C23","panel": "[113, 48, 239, 245]","labels": [[]]}
        ],
        [{"catalysts": [],"coreactants": [],"other species": ["Pd(OAc)2","t-Bu3P","t-BuOK"],
                "temperature": null,"pressure": null,"time": null,"yield": null}
        ],
        [{"smiles": "C1CC2C(C1)N(C3=CC=CC=C23)C4=CC5=C(C=C4)OC(=O)C(=C5)C6=CC=CS6","panel": "[14, 590, 357, 876]","labels": [["548"]]}
        ],
        [{"catalysts": [],"coreactants": [],"other species": ["Pd(OAC)2","(t-Bu)3PHBF4","t-BuOK"],
                "temperature": null,"pressure": null,"time": null,"yield": null}
        ],
        [{"smiles": "C1CC2C(C1)N(C3=CC=CC=C23)C4=CC=CC=C4","panel": "[432, 17, 660, 215]","labels": [["551"]]}
        ],
        [{"smiles": "C1CC2C(C1)N(C3=C2C=C(C=C3)Br)C4=CC=CC=C4","panel": "[421, 284, 680, 512]","labels": [["552"]]}
        ],
        [{"catalysts": [],"coreactants": [],"other species": ["n-BuLi","B(OCH3)3"],
                "temperature": null,"pressure": null,"time": null,"yield": null}
        ],
        [{"smiles": "B(C1=CC2=C(C=C1)N(C3C2CCC3)C4=CC=CC=C4)(OC)OC.C.[S-2].[S-2].[S-2].[Mo]","panel": "[421, 548, 686, 855]","labels":
[["553"]]}
        ],
        [{"smiles": "C1CC2C(C1)N(C3=CC=CC=C23)C4=CC5=C(C=C4)OC(=O)C(=C5)C6=CC=C(S6)C=O","panel": "[20, 891, 367, 1260]","labels": [[]]}
        ],
        [{"catalysts": [],"coreactants": [],"other species": ["CNCH2COOH","CH3COONH4","CH3COOH"],
                "temperature": null,"pressure": null,"time": null,"yield": null
                }
        ],
        [{"smiles": "C1CC2C(C1)N(C3=CC=CC=C23)C4=CC5=C(C=C4)OC(=O)C(=C5)C6=CC=C(S6)/C=C(\\C#N)/C(=O)O","panel": "[2, 1288, 382,
1732]","labels": [["550"]]}
        ],
        [{"catalysts": [], "coreactants": [], "other species": ["NBS","DMF"],
                "temperature": null, "pressure": null, "time": null, "yield": null}
        ],
        [{"catalysts": [],"coreactants": [],"other species": ["Pd(PPh3)4","K2C03/H20"],
                "temperature": null,"pressure": null,"time": null,"yield": null}
        ],
        [{"smiles": "C1CC2C(C1)N(C3=C2C=C(C=C3)C4=CC5=C(C=C4)OC(=O)C(=C5)C6=CC=CS6)C7=CC=CC=C7","panel": "[409, 1128, 816,
1570]","labels": [["554"]]}
        ],
        [{"smiles": "C1CC2C(C1)N(C3=C2C=C(C=C3)C4=CC5=C(C=C4)OC(=O)C(=C5)C6=CC=CS6)C7=CC=CC=C7","panel": "[743, 330, 1153,
770]","labels": []}
        ],
        [{"catalysts": [],"coreactants": [],"other species": ["CNCH2C00H","piperidine"],
                "temperature": null,"pressure": null,"time": null,"yield": [{"Value": 42.0,"Units": "%"}]},
        ],
        [{"smiles": "[HH].C1CC2C(C1)N(C3=C2C=C(C=C3)C4=CC5=C(C=C4)OC(=O)C(=C5)C6=CC=CS6)C7=CC=CC=C7.C1=CC=CC=C1","panel": "[733, 946,
1123, 1397]","labels": []}
        ],
        [{"catalysts": [],"coreactants": [],"other species": ["DMF","POCI3"],
                "temperature": null,"pressure": null,"time": null,"yield": null}
        ],
        [{"catalysts": [],"coreactants": [],"other species": ["DMF","P0Cl1"],
                "temperature": null,"pressure": null,"time": null,"yield": null}
        ]
    ],
    "is_incomplete": false
}
```


Figure S3: A reconstructed reaction graph for the worked example from the main article. The graph is a dictionary, consisting of two keys: 'adjacency', which describes connections between nodes, and 'nodes' which describe all the nodes in the reaction. A node is either an arrow information (reaction conditions) or a set of diagrams, one set per each side of an arrow (step reactants and products).

Image evaluation – challenges

Below, images which our software found particularly challenging in certain areas are presented. These are divided according to the four main detection classes.

Arrow detection

There are no obvious challenges here, but curly arrow detection can probably be further improved.

Diagram detection

Sources of error in diagram detection include:

- Insufficient dilation in postprocessing to cover distant superatoms

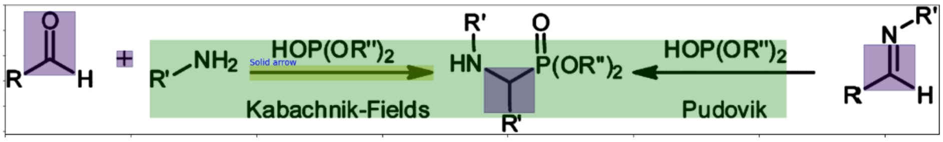

Figure S4: Example image where diagram detection achieved poor accuracy

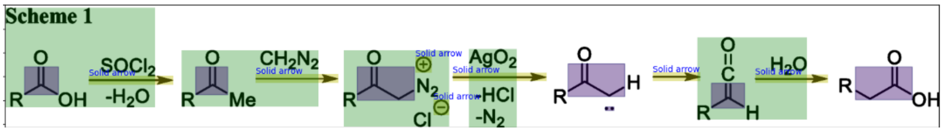

Figure S5: Example image where diagram detection achieved poor accuracy

Label detection

The current challenges are associated with the following scenarios:

- Small elements which are not reflected in the training set are present (e.g. dashes in a dashed line)

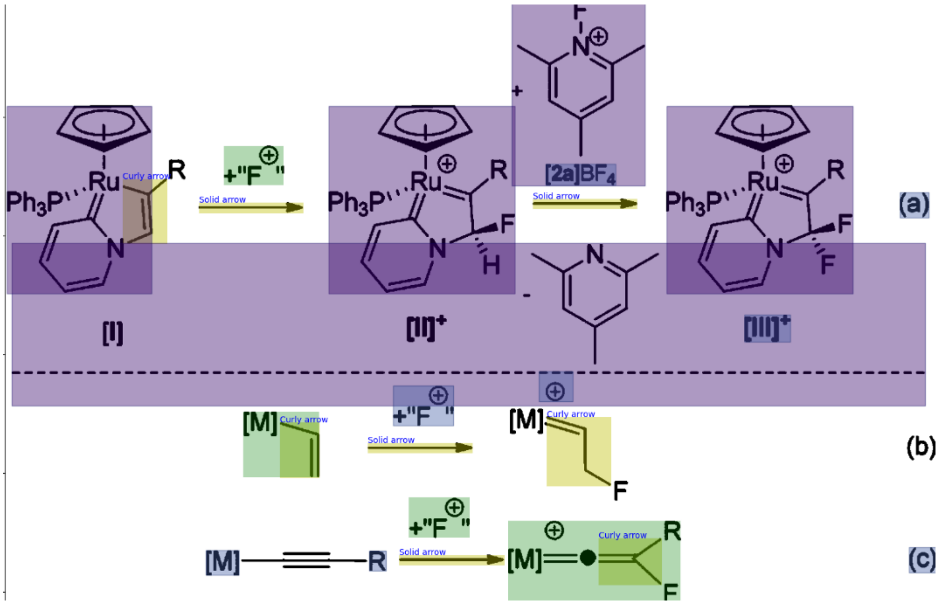

Figure S6: Example image where label detection achieved poor accuracy

- Diagrams of organometallic compounds are present (labels rely on contextual diagrammatic information)

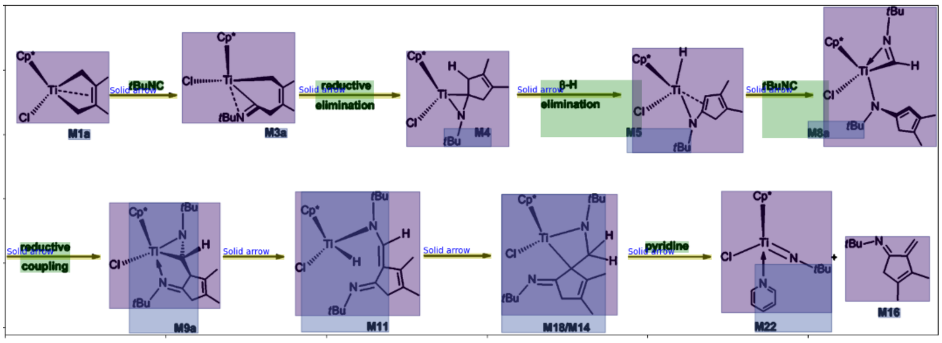

Figure S7: Example image where label detection achieved poor accuracy

Conditions detection

This is currently the most challenging area owing to the relatively small training set. Challenges include the following:

- Auxiliary text present derailing the detection process

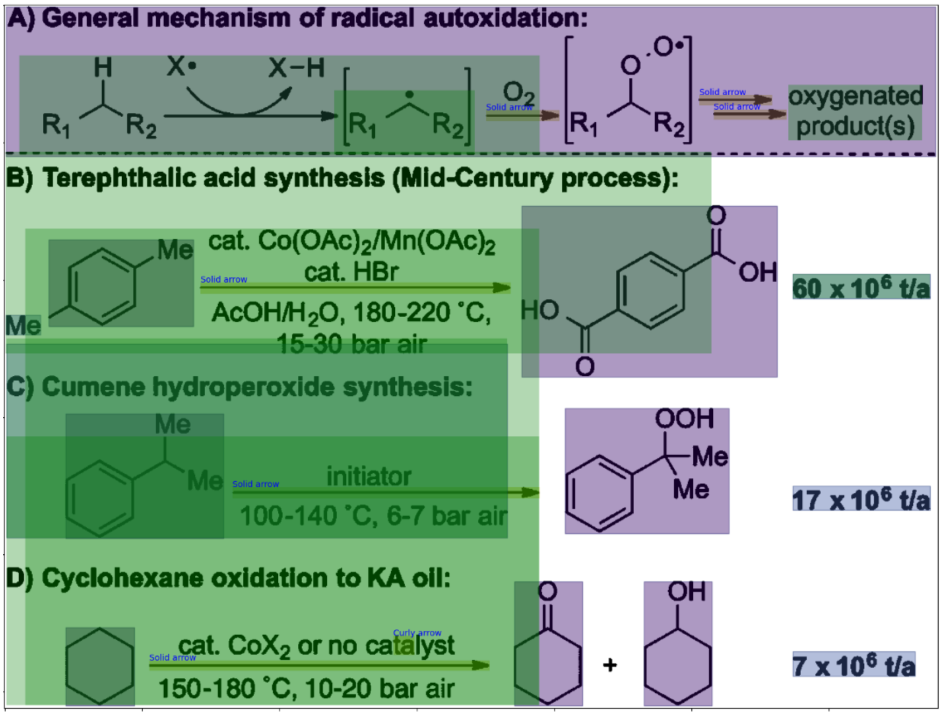

Figure S8: Example image where conditions detection achieved poor accuracy

- Small training set leads to many false positives

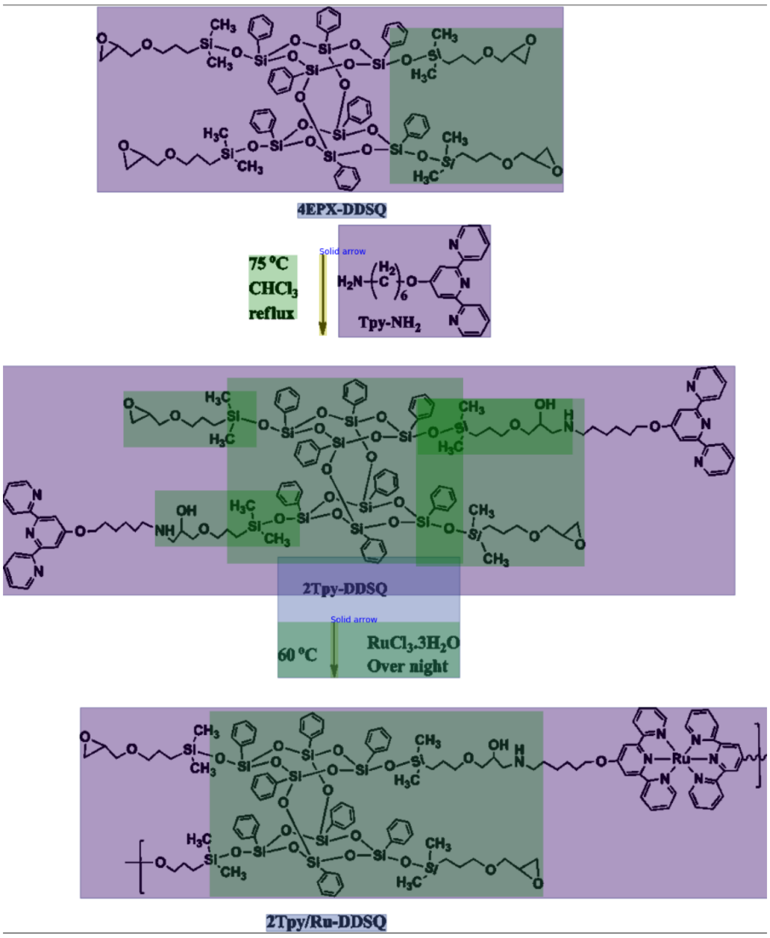

Figure S9: Example image where conditions detection achieved poor accuracy

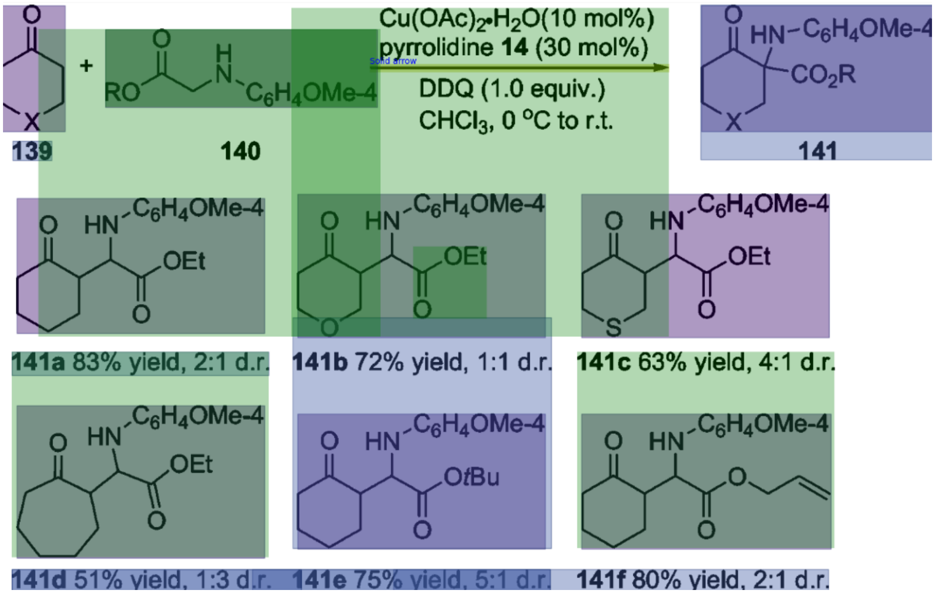

Figure S10: Example image where conditions detection achieved poor accuracy

High level comparison between version 2.0 and 1.0

ReactionDataExtractor has undergone radical changes with respect to version 1.0 to address the most important challenges. Version 1.0 pipeline is given in figure S8, and v. 2.0 workflow is given in figure S11 for reference.

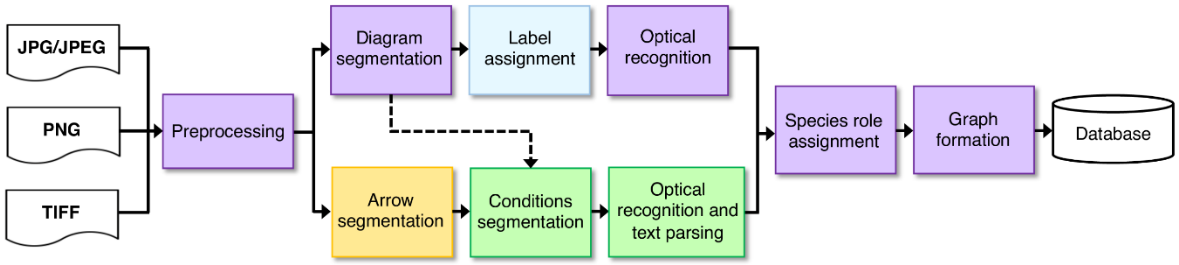

Figure S11: Workflow in ReactionDataExtractor v. 1.0

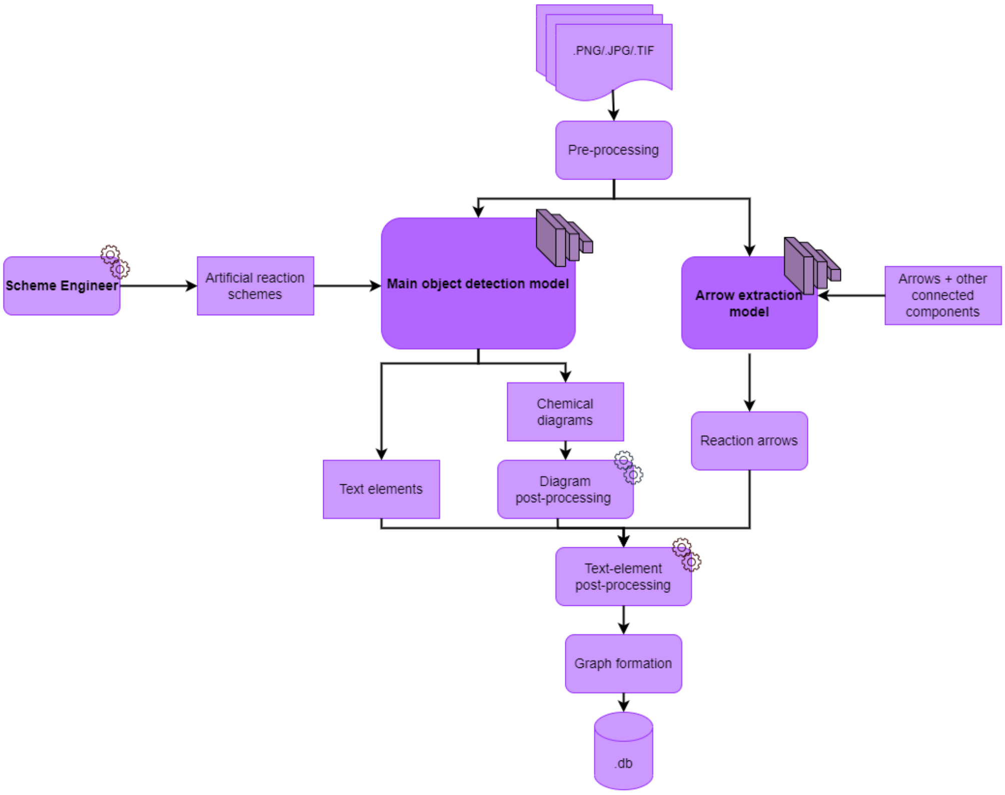

Figure S12. Workflow in ReactionDataExtractor v. 2.0

It should be noted that the term 'segmentation' was replaced with a more accurate term 'detection' in version 2.0.

The main high-level difference concerns a shift in paradigm away from unsupervised machine learning and purely rule-based routines to a data-centric approach combining the latter with deep-learning methods. Combined with a synthetic data generation pipeline, this leads to a much less rigid framework capable of adjusting to fit a particular chemical subdomain of interest.

In terms of the architecture, the main development concerned the structure of extraction modules. In version 1.0 the four extractors (for extracting reaction arrows, chemical diagrams, chemical labels and reaction conditions) are separate entities and operate in two largely independent streams. In the current implementation, the arrow extractor remains separate, but the extraction of the remaining components is handled by a single, unified detection model. The two streams remain independent until the last step concerning text element postprocessing, where information from extracted arrows and diagrams is used to aid their classification.

Removing the rigidity of a rule-based arrow extraction from version 1.0 and delegating the process to a convolutional classifier, allows extraction of all types of arrows. Similarly, replacing unsupervised machine learning models from the pipeline increases flexibility of the pipeline with respect to diagram extraction. When combined, these changes allow for extraction of arbitrary reaction schemes, which was a major limitation of the pipeline in version 1.0

Some low-level functions and methods were adjusted to fit the new domain. This includes the diagram postprocessing routine, where the detected objects are used as inputs to a slightly redesigned routine, as well as the scheme reconstruction which was generalized to account for more complex reaction schemes, as well as cases where some diagrams are situated outside of the main reaction.

Details of the main object detection model

The main object detection model was taken from detectron2 library available at https://github.com/facebookresearch/detectron2 ; this library provides a range of readily available object detection architectures. We used the Faster R-CNN object detector with ResNeXt-101 feature extraction backbone and Feature Pyramid Network (FPN) with the associated configuration file '`faster_rcnn_X_101_32x8d_FPN_3x.yaml`'. We used 2000 synthetically generated reaction schemes to train the object detection model over 5000 iterations via the means of transfer learning using an available pretrained model as a starting point. We used distance intersection-over-union (DIoU) loss for bounding box regression with relative weights of 2.0 and 10.0 for the region proposal stage and the main detection head, respectively; and the default weights for the classification heads. We optimized the neural network using a stochastic gradient descent (SGD) optimizer with a learning rate of 0.001. We used custom anchor square box sizes in the Feature Pyramid Network: [8,16], [16,32], [32,64], [64,128], [256,512] pairs for the 5 scales of the FPN respectively.
